# SUMOylation of annexin A6 retards cell migration and tumor growth by suppressing RHOU/AKT1–involved EMT in hepatocellular carcinoma

**DOI:** 10.1186/s12964-024-01573-2

**Published:** 2024-04-02

**Authors:** Yanfang Yang, Lan Huang, Nan Zhang, Ya-Nan Deng, Xu Cao, Yue Liang, Huijin Hou, Yinheng Luo, Yang Yang, Qiu Li, Shufang Liang

**Affiliations:** 1 Department of Biotherapy, Cancer Center and State Key Laboratory of Biotherapy, West China Hospital, Sichuan University, No.17, 3rd Section of People’s South Road, Chengdu, 610041 People’s Republic of China; 2 Department of Medical Oncology, Cancer Center, West China Hospital, Sichuan University, Chengdu, 610041 China

**Keywords:** Hepatocellular carcinoma, Annexin A6, (de)SUMOylation, Cell migration, Epithelial-mesenchymal transition

## Abstract

**Background:**

The protein annexin A6 (AnxA6) is involved in numerous membrane-related biological processes including cell migration and invasion by interacting with other proteins. The dysfunction of AnxA6, including protein expression abundance change and imbalance of post-translational modification, is tightly related to multiple cancers. Herein we focus on the biological function of AnxA6 SUMOylation in hepatocellular carcinoma (HCC) progression.

**Methods:**

The modification sites of AnxA6 SUMOylation were identified by LC-MS/MS and amino acid site mutation. AnxA6 expression was assessed by immunohistochemistry and immunofluorescence. HCC cells were induced into the epithelial-mesenchymal transition (EMT)-featured cells by 100 ng/mL 12-O-tetradecanoylphorbol-13-acetate exposure. The ability of cell migration was evaluated under AnxA6 overexpression by transwell assay. The SUMO1 modified AnxA6 proteins were enriched from total cellular proteins by immunoprecipitation with anti-SUMO1 antibody, then the SUMOylated AnxA6 was detected by Western blot using anti-AnxA6 antibody. The nude mouse xenograft and orthotopic hepatoma models were established to determine HCC growth and tumorigenicity in vivo. The HCC patient’s overall survival versus AnxA6 expression level was evaluated by the Kaplan–Meier method.

**Results:**

Lys579 is a major SUMO1 modification site of AnxA6 in HCC cells, and SUMOylation protects AnxA6 from degradation *via* the ubiquitin-proteasome pathway. Compared to the wild-type AnxA6, its SUMO site mutant AnxA6^K579R^ leads to disassociation of the binding of AnxA6 with RHOU, subsequently RHOU-mediated p-AKT1^ser473^ is upregulated to facilitate cell migration and EMT progression in HCC. Moreover, the SENP1 deSUMOylates AnxA6, and AnxA6 expression is negatively correlated with SENP1 protein expression level in HCC tissues, and a high gene expression ratio of *ANXA6/SENP1* indicates a poor overall survival of patients.

**Conclusions:**

AnxA6 deSUMOylation contributes to HCC progression and EMT phenotype, and the combination of AnxA6 and SENP1 is a better tumor biomarker for diagnosis of HCC grade malignancy and prognosis.

**Supplementary Information:**

The online version contains supplementary material available at 10.1186/s12964-024-01573-2.

## Introduction

SUMOylation is one of the protein post-translational modifications (PTMs), by which a member of the small ubiquitin-like modifier (SUMO) family of proteins is conjugated to lysine residues in target proteins to regulate multiple cellular processes and biological functions [1]. In the SUMOylation process, the reversible attachment of a SUMO to a protein is controlled by an enzymatic pathway that is analogous to the ubiquitination pathway [2]. In recent years, SUMO modification has become one of the hotspots in the field of life sciences and basic medicine due to a close association of key SUMO-related enzymes or pathways with multiple physiological and pathological regulations. For instance, SAE1/2 is significantly upregulated in cancer tissues of hepatocellular carcinoma (HCC) patients [3], and the survival rate of HCC patients is related to the expression level of SUMO2. In addition, the E2 enzyme Ubc9 is overexpressed in HCC [4]. Our previous study has revealed that SAE1 promotes glioma cancer progression by increasing the SUMOylation and phosphorylation of AKT to involve in relevant molecular signaling pathways, which accelerates the occurrence and development of glioma *in vitro* and *in vivo* [5].

The imbalance between SUMOylation and deSUMOylation of the substrate protein will induce aberrant cell signaling and cell biological activity. For instance, SUMOylation loss dramatically reduces Akt1 E17K-mediated cell proliferation, cell migration and tumorigenesis [6]. We have found that the SUMOylated IQGAP1 is increased in human colorectal carcinoma and IQGAP1 SUMOylation enhances colorectal cancer progression via activating AKT-ERK signaling [7]. These findings indicate the intervention of abnormal protein SUMOylation possibly represents a potential therapeutic approach for treating cancer.

Annexin A6 (AnxA6) is a calcium-dependent phospholipid-binding membrane protein that belongs to the conserved annexin protein family with two duplicative core annexin modules. AnxA6 is predominantly located in cell plasma membrane or endosomal compartment to recruit signaling proteins, modulate cholesterol and membrane transport, actin dynamics and vesicle fusion in secreting epithelia during exocytosis [8, 9]. Hence, AnxA6 has been implicated in many biological processes, closely associated with a variety of cancers.

AnxA6 seems to have variable even opposite roles in multiple tumors. It has been implicated as a potential marker for cervical cancer [10], but as a tumor suppressor in cervical cancer [11], gastric cancer [12], prostate cancer [13] and others. AnxA6 exerts a promoting factor in cellular adhesion, motility and invasiveness of breast cancer [14, 15], and the progression of acute lymphoblastic leukemia [13]. Numerous previous studies have focused on AnxA6 biological role by tracking protein expression profiling with cancers. However, the PTMs of AnxA6 are rarely understood in cancers so far.

Our recent study has demonstrated that AnxA6 is SUMOylated with SUMO1 conjugation at lysine (K) 299 in epithelial cancer, but the biological function of AnxA6 SUMOylation in HCC is unclarified [16]. Herein, we have revealed that AnxA6 deSUMOylation promotes cell migration and HCC progression. AnxA6 downregulation in HCC is associated with the downregulated SUMOylation level of AnxA6, and the SUMO1 modification of AnxA6 at the residue K579 stabilizes AnxA6 protein itself to counteract ubiquitin-mediated degradation. Moreover, the deSUMOylated AnxA6 enhances cell migration and tumor development of HCC *in vitro* and *in vivo*. Therefore, it is pivotal to exert multiple biological functions for AnxA6 not only through interacting with other partners but also depending on its specific SUMOylation and deSUMOylation levels in HCC.

## Materials and methods

### Antibodies

The primary antibodies included AnxA6 (sc-166807) and N-cadherin (sc-31031) were ordered from Santa Cruz Company, SUMO1 (ET1606-53), ubiquitin (ET1609-21), AKT1(ET1609-47), p-AKT1^ser473^(ET1607-73), vimentin (ET1610-39), E-cadherin (EM0502), twist (RT1635) and RHOU (ER1918-73) were ordered from HuaBio Company in China. AnxA6 (A5390) was ordered from ABclonal. The antibody β-actin (TA-09, Zsbio) was used to quantify the expression of housekeeping gene β-actin for comparison normalization.

### HCC tissue samples

This study was approved by the Institutional Ethics Committee of State Key Laboratory of Biotherapy, West China Hospital of Sichuan University. 15 pairs of HCC tissues and adjacent tissues were surgically resected to collect in West China Hospital, Sichuan University (Chengdu, P. R. China). Each case was identified through pathologic biopsy. The clinical characteristics of the enrolled subjects were summarized in Supplementary Table S1.

A commercial tissue array with 38 HCC tissues (OD-CT-DgLiv02-004) was ordered from Shanghai Outdo Biotech Co., Ltd (Shanghai, China) to measure the protein expression of AnxA6. The clinicopathological data included patient age, gender and pathological stage.

### Cell culture

HEK293T, normal liver cell line LO2 and several human HCC cell lines, including HepG2, SK-Hep1, Hep3B and HepG2.2.15, have been stored in our laboratory. Human HCC cell lines MHCC-97 H and MHCC-97 L have been established in the Liver Cancer Institute of Fudan University, and these cell lines are generously endowed for our research [17].

MHCC-97 H and MHCC-97 L cells are originally derived from the same tumor with a spontaneous pulmonary metastasis occurred in 100% and 40% [18]. Hepa 1–6 is a murine hepatoma cell line derived from the BW7756 hepatoma tumor and is inoculated in the C57BL/6J mouse for* in vivo* experiment testing. These cells are cultured in DMEM (HyClone) containing 10% FBS (HyClone) and maintained in a humidified 5% CO_2_ incubator at 37 °C. The cell lines were authenticated using short tandem repeat (STR) analysis.

### 12-O-tetradecanoylphorbol-13-acetate (TPA) treatment

LO2 and HepG2 cells were respectively induced into the epithelial-mesenchymal transition (EMT)-featured cells by 100 ng/mL TPA exposure, which was described in our previous paper [19]. The EMT markers were monitored during TPA inducement respectively for 0, 4, 8 and 12 days. The stable EMT cells were acquired from TPA-induced LO2 and HepG2 cells with EMT features by continuous TPA stimulation in cell passage process. The EMT cells usually have high cell migration ability.

### Cell migration assays

Cell migration ability was examined using a 24-well transwell plate with 8 mm pore polycarbonate membrane inserts (Millipore) [7, 20]. After treatment or transfection, 1 × 10^5^ cells/well HCC cells were seeded in serum-free medium in the top chambers of a transwell plate for 24 h, cells that were attached to the upper surface of the membrane were carefully removed with cotton swabs, fixed with 4% paraformaldehyde for 25 min at room temperature and stained with 0.1% crystal violet for 15 min. Images were captured with a microscope, and three random fields were selected to count the number of migration cells.

### Plasmids and cell transfection

AnxA6 cDNA (gi71773329) was cloned into a eukaryotic expression vector pTango-zeo-N4-Flag, and the recombinant plasmid pFlag-AnxA6 was obtained and verified by DNA sequencing. The mutant plasmids, including pFlag-AnxA6^K75R^, pFlag-AnxA6^K306R^, pFlag-AnxA6^K418R^ and pFlag-AnxA6^K579R^, were derived from pFlag-AnxA6 through site direct mutagenesis, which was confirmed by DNA sequencing.

The plasmids pHis-SUMO1, pMyc-SUMO1, pHA-Ubc9 and pHA-ubiquitin were maintained in our laboratory [7]. Plasmids were respectively transiently transfected into HEK293T, HepG2 and other cell lines with the transfection reagent (Lipofectamine2000, 11,668–019, Life Technologies) to observe biological effects.

To generate stable AnxA6-overexpressing HCC cells, the plasmid pLV203-AnxA6 or pLV203-AnxA6^K579R^ was constructed to overexpress AnxA6 by a lentivirus delivering system containing other two plasmid package components of psPAX2 and pMD2.G. These three plasmids were transfected into HEK293T cells to collect supernatants after transfection for 48–72 h, and the lentivirus supernatants carrying pLV203-AnxA6 or pLV203-AnxA6^K579R^ were used to infect HCC cells for 4–6 days.

### Immunoprecipitation (IP) & western blotting

For the IP experiment, cell lysate was incubated with 50 μL of slurry anti-Flag M2 affinity gel (A2220, Sigma) to enrich Flag-tag proteins. Meanwhile, another part of cell lysate was incubated with the target antibody coupled with the protein A beads (161–4013, Bio-Rad) overnight at 4 °C to enrich AnxA6-interacting proteins [5, 7]. As a negative control, the primary antibody was replaced with the normal rabbit IgG (A7016, Beyotime) to eliminate the nonspecific binding protein. The enriched proteins were subjected to isolation on SDS-PAGE to test the target protein by western blotting.

For western blotting, protein samples from cell lysates or immunoprecipitation (IP) were separated by 7.5–10% SDS-PAGE gel, and proteins were then transferred onto PVDF membranes (Millipore, Billerica, MA). Membranes were then blocked with 5% nonfat milk, incubated with the primary antibody overnight at 4 °C, and then incubated with horseradish peroxidase–linked secondary antibody for 1 h at room temperature. After adding ECL reagents, protein signals were detected with a luminescent image analyzer. The density of the target band in western blot was measured with Image J software for semi-quantification of staining signals.

### Half-life assay of AnxA6 protein

Plasmid pFlag-AnxA6 or pFlag-AnxA6^K579R^ was transfected to HepG2 cells for 48 h, then cells were treated with 100 μg/mL cycloheximide (CHX) for the indicated times. Cell extracts from each time point were resolved by SDS-PAGE and detected by western blotting using anti-Flag (M1403-2, HuaBio).

### Cell immunofluorescence & immunohistochemistry

Cell immunofluorescence [19] was performed to observe protein cellular distribution. Briefly, cells were fixed with 4% paraformaldehyde, blocked in 5% goat serum and 0.25% Triton X-100, and incubated with indicated primary antibodies overnight. Cells were then rinsed in PBS three times and incubated with appropriate fluorescence-conjugated secondary antibodies. After washing with PBS three times, cells were incubated with DAPI. The images were obtained using a Zeiss LSM880 confocal microscopy. The Integrated Density (IntDen) values, which was the mean grey value multiplied by the area of the cell, for further analysis.

The protein expression level of AnxA6, SENP1, RHOU and p-AKT1^ser473^ in HCC tissues was detected by immunohistochemistry (IHC) following our previous approaches [5, 7]. The IHC scores for each tissue sample, ranging from 0 to 12, were measured as immunostaining intensity multiplied by the percentage of positive cells. An immune-reactivity score of less than 4 was defined as a low expression level, and a score of more than 4 was defined as a high expression.

### Subcutaneous HCC xenograft model

The mouse xenograft experiments were approved and conducted by the Institutional Animal Care and Treatment Committee of Sichuan University in China. Five-week-old male BALB/c nude mice (HFK bioscience, Beijing, China, Strain number: D000521) were randomly assigned into two groups. The experimental group was injected with stable AnxA6-overexpressing HepG2 cells to observe tumor growth (*n* = 5), and the control group was injected with HepG2 cells (*n* = 5). The experimental group was subcutaneously injected with 5 × 10^6^ stable AnxA6-overexpressing HepG2 cells in 0.1mL serum-free DMEM into the right flank of each mouse, whereas the control group was injected with the same quantity of HepG2 cells. Approximately 4 weeks later, tumors were harvested to monitor size and detect protein expression [7, 19]. During the experiment progression, the dying mice were excluded from the analysis. Totally 5 mice in the experimental group, and 5 mice in the control group entered the result analysis.

### Mouse orthotopic hepatoma model

Hepa 1–6 cells were orthotopically injected in the liver of 6-8-weeks-old male C57BL/6J mice (HFK bioscience, Beijing, China, Strain number: N000013) to establish the hepatoma model, which was used to observe AnxA6 influence on tumor growth and detect AnxA6-regulated signaling pathway in vivo. Briefly, after anesthesia and median laparotomy, the surface of the median liver lobe was exposed to inject 20–30 μL of 1 × 10^6^ Hepa 1–6 cells or AnxA6-overexpressing Hepa 1–6 cells (*n* = 3 for each group) in the subcapsular region within the liver of a C57BL/6J mouse. After injection for 14 days, mice were dissected to observe the liver tissues. Tumors were located diffusely within the mouse liver with a large area, where the brown part was liver tissue and the white part was tumor tissue.

### Liquid chromatography with tandem mass spectrometry (LC-MS/MS)

Cells overexpressing AnxA6 and SUMO1^T95K^, were collected to enrich SUMO1-tagging AnxA6 by IP, then the target band was separated on SDS-PAGE. The protein band was cut to digest by Lys-C enzyme, and peptides were identified by liquid chromatography-tandem mass spectrometry (LC-MS/MS) on an easy nano-LC1000 HPLC system (Thermo Scientific, San Jose, CA) and a Q-Exactive™ Plus hybrid quadrupole-Orbitrap MS (Thermo Scientific, San Jose, CA), which was mainly described as before [21, 22].

The MS/MS conditions were included as follows. The survey scan ranged from 300 to 1800 m/z at a resolution of 70,000. After a full scan, the top 10 MS fragments were selected for higher-energy collisional dissociation (HCD). The isolation window was acquired at a resolution of 17,500 with an isolation window of 1.6 m/z. The MS/MS scan was 1 × 10^6^ with a maximum injection time of 20 ms, and that for the MS/MS scan was 1 × 10^5^ with a maximum injection time of 100 ms.

The data were searched by the MaxQuant search engine (version 2.2, Matrix Sciences, London, UK) with the following parameters, including the human UniProt database (version 2021.04), up to two missed cleavage sites for Lys-C, peptide mass tolerance of 7 ppm, and fragment mass tolerance of 0.5 Da for HCD. Carbamidomethylation of cysteine was specified as a fixed modification, whereas oxidation (M), acetyl (protein N-term), and KGG (only for indicating SUMOylation) were defined as variable modifications. The false discovery rates (FDRs) of peptide and protein were set to 0.01 FDR. And at least one unique peptide of a protein successfully detected was acceptable.

### Database and bioinformatics analysis

The gene transcriptional levels of *RHOU* and *SENP1* in HCC tissues and adjacent normal samples were analyzed from online Gene Expression Profile Interactive Analysis database (GEPIA) database (http://gepia.cancer-pku.cn/). The gene level correlation between *AnxA6* and *SENP1* was analyzed in GEPIA and cBioPortal (https://www.cbioportal.org/) database. The Kaplan–Meier plotter (http://kmplot.com/analysis/) was used for *AnxA6/SENP1*-based survival analysis of HCC patients.

### Statistical analysis

Data were analyzed using the two-tailed Student’s t-test in GraphPad Prism 8.0 (GraphPad Software). Bar graphs represented the mean of independent experimental repeats unless otherwise stated. Two-tailed unpaired student’s t-test was used for comparing two groups of data. One-ANOVA was used to compare multiple groups of data. Statistical significance representations: *, *P* < 0.05; **, *P* < 0.01; ***, *P* < 0.001.

## Results

### AnxA6 overexpression retards HCC cell migration and tumorigenesis

**Fig. 1:**
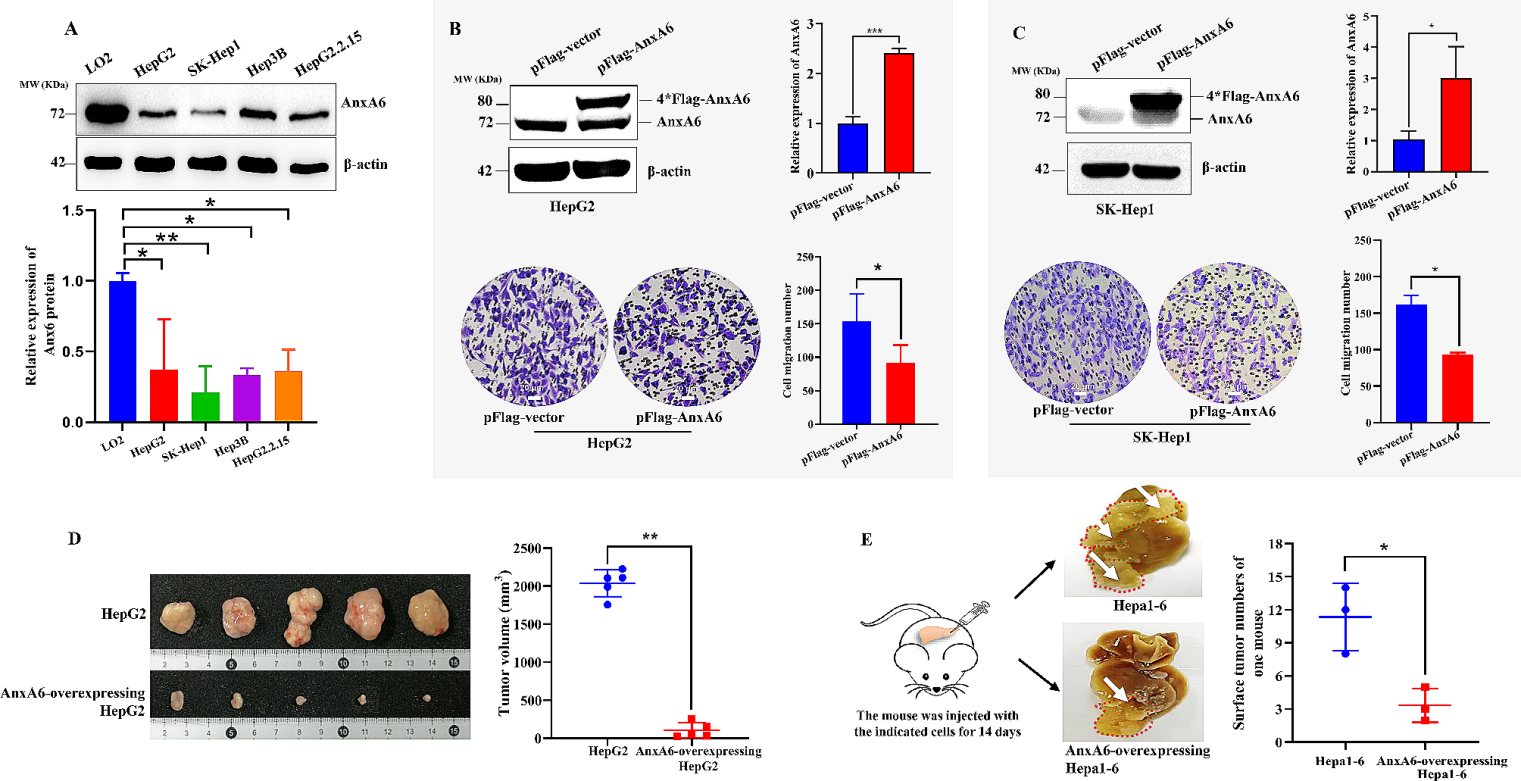
AnxA6 overexpression retards cell migration and tumorigenesis. (**A**) The protein expression of AnxA6 was detected in LO2 and several HCC cell lines by western blotting (up). β-actin was used as an internal control. The quantitative data of AnxA6 expression in LO2 and HCC cells were the means ± SD from three independent experiments (bottom). (**B**-**C**) AnxA6 overexpression inhibited cell migration of HepG2 or SK-Hep1. The plasmid pTango-4*Flag-AnxA6 (pFlag-AnxA6) was transiently transfected into HepG2 or SK-Hep1 for 48 h, then cell migration was analyzed by the transwell assay for another 24 h of culture. Images were captured with a microscope, and three random fields were selected to count the number of migration cells. Scale bar represented 20 μm. (**D**-**E**) AnxA6 overexpression suppressed mouse tumor growth of the subcutaneous HCC xenograft (**D**) and orthotopic hepatoma models (**E**). For subcutaneous HCC xenograft models, 1 × 10^7^ HepG2 or AnxA6-overexpressing HepG2 cells, were subcutaneously injected into each nude mouse with male BALB/c individually, and the tumors in each group (*n* = 5) were collected to weigh after 4 weeks (**D**). (**E**) In the mouse orthotopic HCC model that was injected with wild-type murine Hepa1-6 cells or AnxA6-overexpressing Hepa 1–6 cells, the liver tumors of one mouse were indicated with red dotted line and white arrow (left), and the mean numbers of liver surface tumors respectively from 3 mice were counted (right). ns, no statistical; *P < 0.05; **P < 0.01; ***P < 0.001

Firstly, we compared the protein expression of AnxA6 in several HCC cell lines by western blotting. Compared with the normal liver cell line LO2, the AnxA6 protein had a significantly lower expression level in several HCC cell lines, including HepG2, SK-Hep-1, Hep3B and HepG2.2.15 (Fig. 1A). Based on a relatively low endogenous protein level of AnxA6 in HepG2 and SK-Hep-1 cells, we transiently transfected plasmids pTango-4Flag-AnxA6 (pFlag-AnxA6, ∼ 80 KDa) into these cells to study influence of AnxA6 overexpression on HCC cell migration ability. A transwell assay results showed that the ectopic expression of AnxA6 significantly decreased the number of migrated HepG2 cells after transfection for 48 h (*p* < 0.05) (Fig. 1B, bottom). A similar result was obtained in SK-Hep-1 cells (*p* < 0.05) (Fig. 1C, bottom). These results demonstrate AnxA6 overexpression retards cell migration.

Furthermore, the *AnxA*6 cDNA sequence was inserted into a lentivirus vector that was used to screen stable AnxA6-overexpressing HepG2 and Hepa1-6 cells. The anti-tumor effect of AnxA6 in xenograft or orthotopic mouse models was evaluated. 5 × 10^6^ stable AnxA6-overexpressing HepG2 cells were inoculated into the right flank of nude mice (*n* = 5 for each group). After cell injection four weeks later, the average tumor volume of AnxA6-overexpressing mice with 105.25 mm^3^ was 19.35 times smaller than the volume with 2036.42 mm^3^ of the control group inoculated with HepG2 cells (Fig. 1D, *p* < 0.05), which shows that AnxA6 overexpression slows down tumor growth in mouse HCC xenograft models.

A similar result was verified in an orthotopic hepatoma model by injecting Hepa1-6 cells. The stable AnxA6-overexpressing Hepa1-6 cells were respectively injected into C57BL/6J mouse liver, and after injection for 14 days, the mean numbers of the surface tumors (*n* = 3) for a mouse injected by AnxA6-overexpressing Hepa1-6 were significantly less than that of Hepa1-6-injecting group (*n* = 11) (Fig. 1E, *p* < 0.05). In general, AnxA6 overexpression retards mouse liver tumor growth.

### AnxA6 inhibits HCC cell migration via weakening RHOU/AKT1-involved EMT

**Fig. 2:**
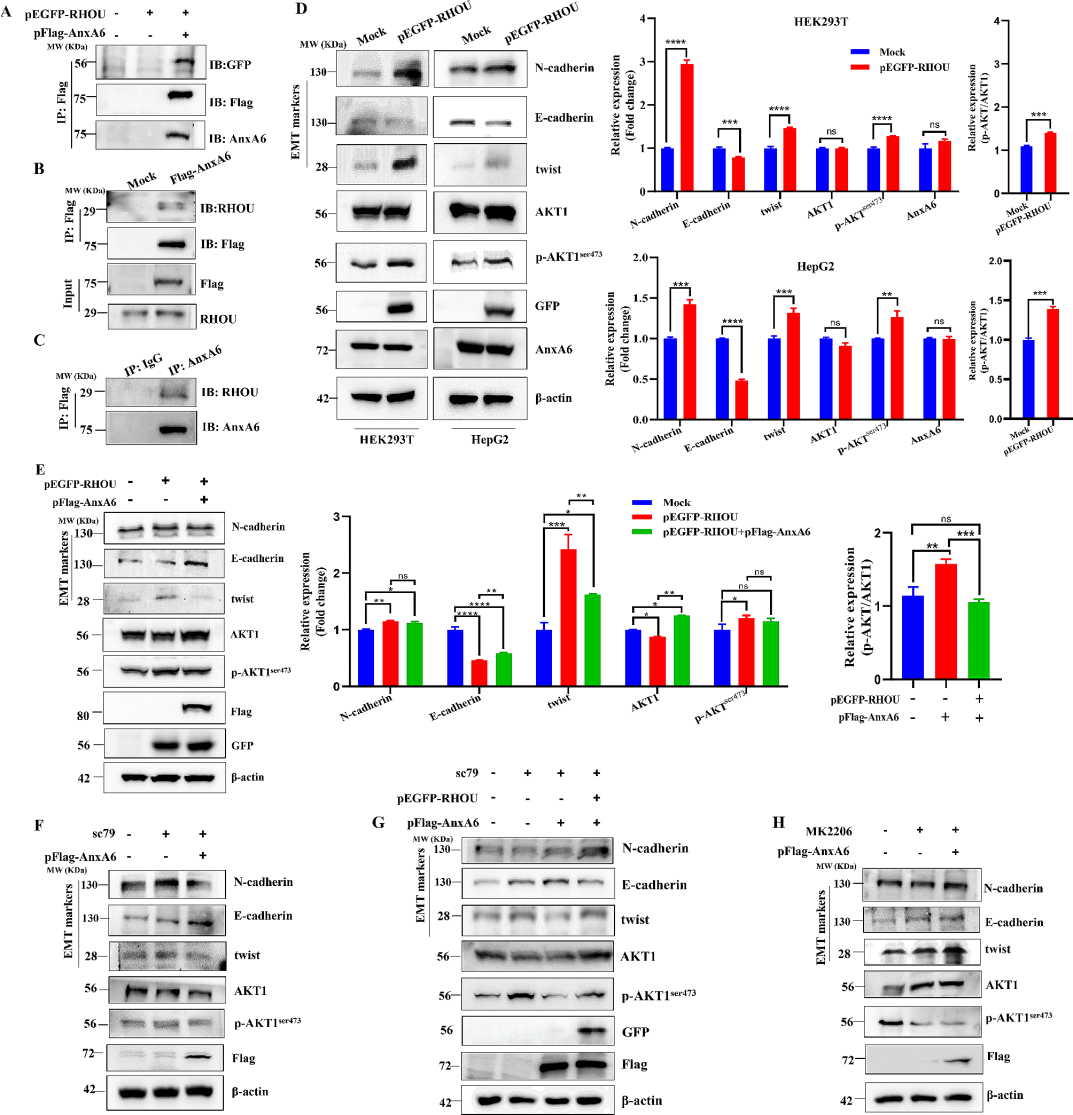
AnxA6 inhibits HCC cell migration via weakening RHOU/AKT1-involved EMT. (**A**) Ectopic expression of AnxA6 was confirmed to interact with GFP-tagging RHOU protein. Co-transfection with plasmids pEGFP-RHOU and pFlag-AnxA6 into HepG2 cells, then Flag-tagging AnxA6 was enriched by co-IP, and the AnxA6-interacting RHOU was detected in protein precipitation using anti-GFP antibody. (**B**) Ectopic expression of AnxA6 was confirmed to interact with endogenous RHOU protein. HepG2 cells were transfected with pFlag-AnxA6 plasmids for 48 h, the lysates were performed IP to capture AnxA6 and subsequently immunoblotted with RHOU antibody. (**C**) Endogenous AnxA6 interacts with RHOU. The lysates of HepG2 cells were performed IP to capture endogenous AnxA6 and subsequently immunoblotted with RHOU antibody. (**D**) Protein expression level and quantification analysis of EMT markers and p-AKT1^ser473^ in HEK293T and HepG2 cells. HEK293T and HepG2 cells were transiently transfected with pEGFP-RHOU for 48 h, and then several key EMT markers and p-AKT1^ser473^ were detected. (**E**) AnxA6 overexpression inhibited cell migration *via* retarding RHOU-AKT1 signaling pathway. Plasmid pEGFP-RHOU was transiently transfected with or without pFlag-AnxA6 into HepG2 cells for 48 h, and EMT markers and p-AKT1^ser473^ were detected in the indicated groups (left). Data were represented as the mean ± SD of three separate experiments (right). ns, no statistical; *P < 0.05; **P < 0.01; ***P < 0.001. (**F**) HepG2 cells were transfected with pFlag-AnxA6 plasmids and treated with 5μM AKT activator SC79 for 24 h to measure EMT markers. (**G**) HepG2 cells were transiently transfected pFlag-AnxA6 with pEGFP-RHOU for 48 h and treated with 5μM AKT activator SC79, then several key EMT markers and p-AKT1^ser473^ were detected. (**H**) HepG2 cells were transfected with pFlag-AnxA6 plasmids and treated with 10μM inhibitor MK2206 for 24 h to measure EMT markers

The interacting protein network with AnxA6 was analyzed from the BIOGRID database (https://thebiogrid.org) to screen the potential target signaling proteins recruited by the scaffold protein AnxA6 in cell migration. As a result, the Ras homolog family member U (RHOU) is predicted to interact with AnxA6, which has been reported to positively correlate with cell migration [23, 24]. Then the interaction between AnxA6 and RHOU was validated by a co-immunoprecipitation (co-IP) experiment. The plasmids pFlag-AnxA6 and pEGFP-RHOU were co-transfected into HepG2 cells and enriched the cellular exogenous AnxA6 and its binding proteins using immunoprecipitation against Flag antibody, and the EGFP-tagging RHOU was detectable by western blot using GFP antibody in the enriched proteins. The interaction was validated by co-IP respectively using the ectopic Flag-tagging AnxA6 and EGFP-tagging RHOU (∼ 56 KDa) in HepG2 cells (Fig. 2A, Supplementary Fig. 1A). We also confirmed that the endogenous 29 KDa RHOU protein interacted with endogenous AnxA6 after IP enrichment in HepG2 cells (Fig. 2B-C, Supplementary Fig. 1B-C).

To investigate the roles of AnxA6 and RHOU in cell migration, the plasmids pFlag-AnxA6 and pEGFP-RHOU were co-transfected into cells to compare with the expression of biomarker proteins related to the epithelial-to-mesenchymal transition (EMT). When transfection of a single type plasmid pEGFP-RHOU, RHOU overexpression would lead to expression change of several EMT biomarkers, including an increase of N-cadherin and twist, while decrease of E-cadherin. Meanwhile, the phosphorylation of AKT (p-AKT1^ser473^) was significantly increased in HEK293T cells (Fig. 2D, Supplementary Fig. 1D), which has been reported to involve in EMT process [25]. A similar result was obtained in HepG2 cells (Fig. 2D, Supplementary Fig. 1D).

We further detected AnxA6 influence on the RHOU-mediated EMT process. Compared with a single plasmid transfection of pEGFP-RHOU (Fig. 2E, lane 2), the additional pFlag-AnxA6 transfection weakened the expression of twist and p-AKT1^ser473^ (Fig. 2E, lane 3) in HepG2 cells. Moreover, the overexpression of AnxA6 led to twist downregulation and E-cadherin upregulation in HepG2 cells under 5 μM AKT activator SC79 treatment for 24 h (Fig. 2F). However, overexpression of RHOU attenuated the function of AnxA6 in the EMT process (Fig. 2G, lane 3). In addition, we treated HepG2 with 10 μM AKT inhibitor MK2206 for 24 h, the ability of AnxA6 protein suppressing AKT-involved EMT processes was deprived due to AKT inhibitor intervention, and there was no significant expression difference in EMT markers (Fig. 2H). This indicated that AnxA6 protein affected the EMT process through the p-AKT1 signaling. Taken together, these results support AnxA6 inhibits cell migration via weakening RHOU/AKT1-involved EMT process.

### AnxA6 level is downregulated in EMT-featured cells

**Fig. 3:**
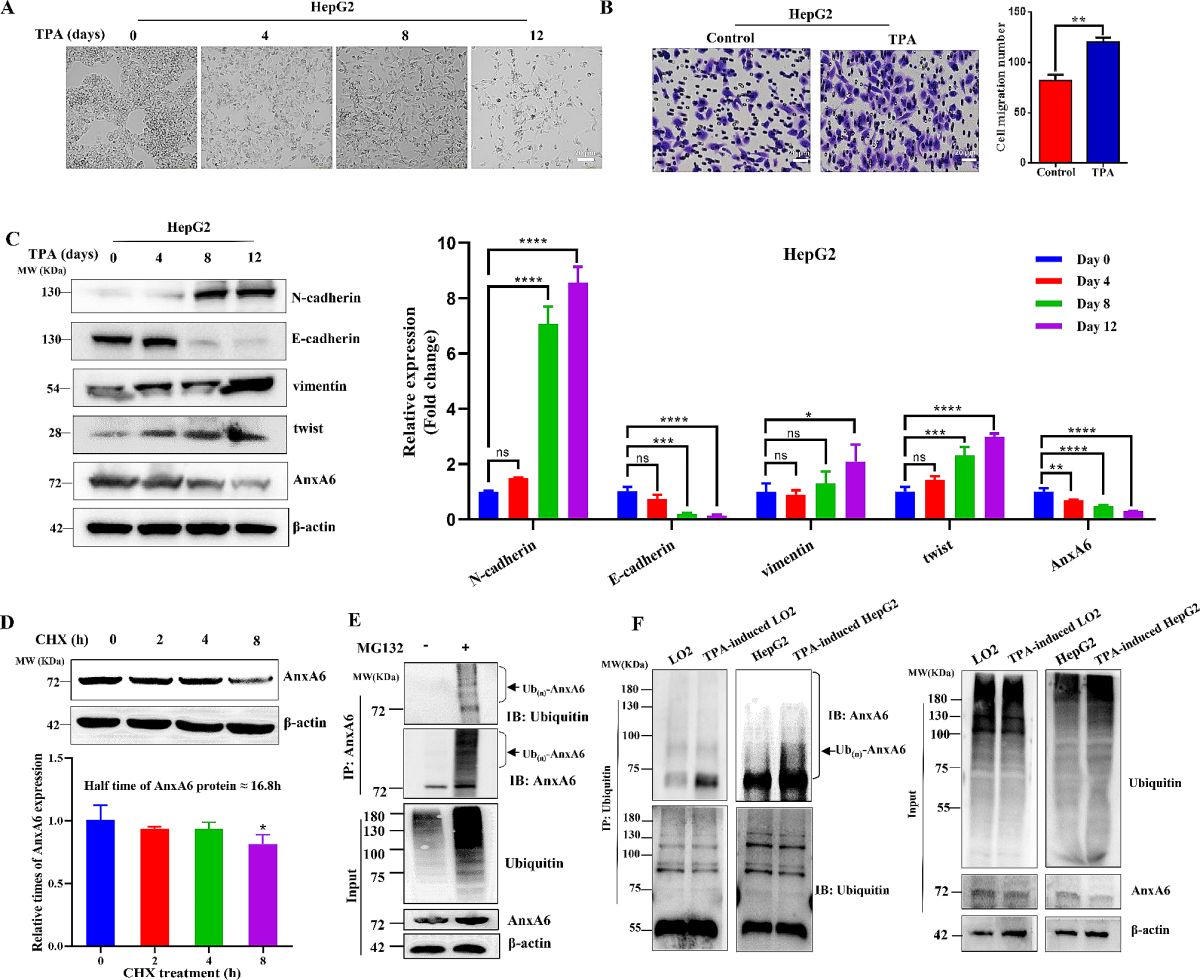
Ubiquitin-mediated AnxA6 degradation in EMT cells. (**A**) Cell morphology changes of HepG2 cells under TPA exposure. HepG2 cells were treated with 100 ng/mL TPA for 0, 4, 8, 12 days, and the images were captured with a microscope. Scale bars, 20 μm. (**B**) Cell migration ability was increased in TPA-induced EMT cells. Cell migration of the TPA-induced HepG2 cells was analyzed using transwell assays. Images were captured with a microscope, and three random fields were selected to count the number of migration cells. *p < 0.05, **p < 0.01. Scale bars, 20 μm. (**C**) EMT biomarkers were dynamically expressed in TPA-induced cells at different treatment days. HepG2 cells were treated with 100 ng/mL TPA for 0, 4, 8, 12d, and cell proteins were collected for western blot with indicated antibodies (left). Quantification of protein levels of EMT markers in TPA-induced HepG2 cells. Data were represented as the mean ± SD of three separate experiments (right). ns, no statistical; *P < 0.05; **P < 0.01; ***P < 0.001. (**D**) Cell endogenous AnxA6 protein level was a time-dependent decrease in response with cycloheximide (CHX) incubation. The HepG2 cells were incubated with 100 μg/mL CHX for 0, 2, 4 and 8 h, then cell lysates were used to detect target protein level with anti-AnxA6 and anti-β-actin antibodies. *P < 0.05. (**E**) AnxA6 was validated to degrade *via* ubiquitination in HepG2 cells under 20 μM MG132 exposure for 12 h. Cell lysates were extracted to concentrate AnxA6 by IP and detected by anti-AnxA6 or anti-ubiquitin antibodies. (**F**) The levels of AnxA6 ubiquitination were increased in EMT-featured cells. The EMT-featured cells were treated with 20 μM MG132 for 12 h, then the lysates were IP to capture ubiquitin protein and analyzed by immune-blotting with AnxA6 antibody. Ub_(n)_-AnxA6, Ubiquitinated AnxA6

The carcinogenic agent 12-O-tetradecanoylphorbol-13-acetate (TPA) can induce obvious cellular phenotypic changes and lead to EMT phenotype [19]. After 100 ng/mL TPA treatment, HepG2 cells were stepwise changed from cobblestone-like to spindle-like shapes (Fig. 3A). Cell migration of EMT-featured HepG2 cells was significantly higher than the untreated parent cells (*p* < 0.05) (Fig. 3B). Moreover, the EMT markers showed a time-dependent change, including a considerable increase in N-cadherin, vimentin and twist, while downregulation of AnxA6 and E-cadherin (Fig. 3C). A similar result was obtained in LO2 cells (Supplementary Fig. 2). Taken together, these data indicate AnxA6 level is decreased in EMT-featured cells, however the molecular mechanism responsible for the outcome is unclear.

We investigated the mechanism of AnxA6 downregulation in EMT cells, whether it is related to protein degradation by ubiquitin or small ubiquitin-like modification. Our finding of AnxA6 decrease in EMT was consistent with the previous study on AnxA6 role in cancer cell migration [26, 27]. Firstly, we analyzed AnxA6 protein level change under cellular newborn protein translation arrest with cycloheximide (CHX) treatment. After HepG2 cells were incubated with 100 μg/ml CHX for 0, 2, 4 and 8 h, the AnxA6 protein level was detected by western blot. The halftime of AnxA6 was 16.8 h, and about a 20% reduction of endogenous AnxA6 was observed in response to CHX treatment for 8 h (Fig. 3D).

The previous report that AnxA6 has several ubiquitination sites identified by the proteomics method [28, 29]. We further explored whether AnxA6 reduction was dependent or independent of the ubiquitin proteasome-regulated degradation. IP and Western blotting demonstrated that the cellular AnxA6 was confirmed to have an obvious ubiquitin-modified pattern after HepG2 cells were incubated with 20 μM MG132 for 12 h. The endogenous 68 KDa AnxA6 was visible along with multiple protein bands respectively in AnxA6 antibody-enriched protein samples (Fig. 3E, Lane 2). We examined the degree of AnxA6 ubiquitination and AnxA6 expression in TPA-induced EMT cells. The level of ubiquitinated AnxA6 was increased in EMT cells after enriching ubiquitin by IP (Fig. 3F), which contributes to AnxA6 degradation through the ubiquitin-proteasome system.

### SUMOylation stabilizes AnxA6 to counteract ubiquitin-mediated protein degradation

**Fig. 4:**
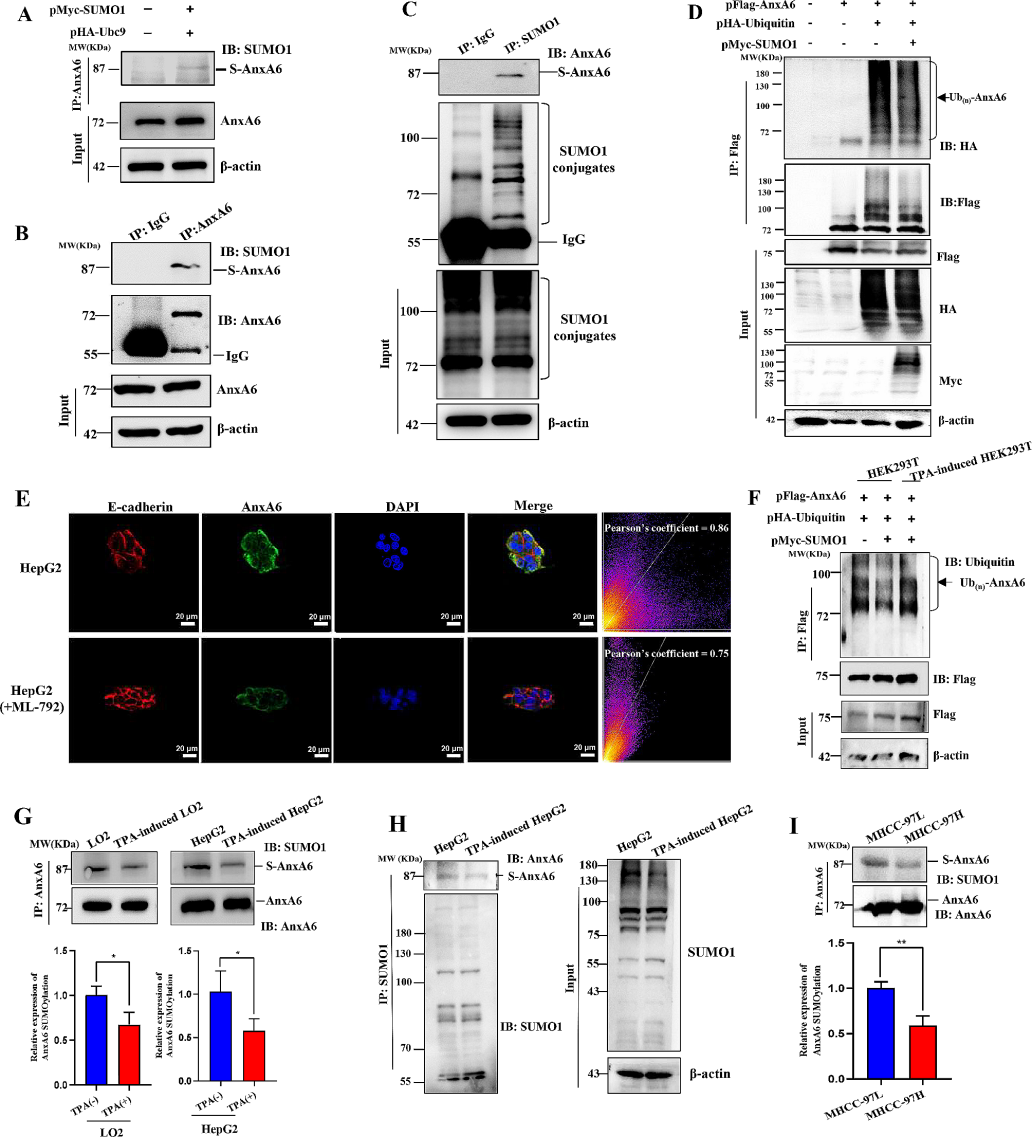
SUMOylation stabilizes AnxA6 protein to counteract ubiquitin-mediated degradation. (**A**) The SUMO1-modified AnxA6 was detected in HEK293T cells under co-transfection of plasmids pHA-Ubc9 and pMyc-SUMO1. The SUMOylated AnxA6 was captured by IP with anti-AnxA6 in HEK293T cells. (**B**-**C**) The endogenous SUMOylated AnxA6 was measured in HEK293T cells. The SUMOylated AnxA6 was captured by IP with anti-AnxA6 (**B**) or anti-SUMO1 (**C**) antibody, then respectively immunoblotted with anti-SUMO1 or anti-AnxA6 antibody. The normal IgG was a nonspecific binding control for IP. S-AnxA6: Flag-tagging SUMOylated AnxA6. (**D**) Antagonism between SUMOylation and ubiquitination of AnxA6 protein. After the target plasmids were transfected into HepG2 cells for 48 h, 20 μM MG132 as a proteasome inhibitor was added to incubate with cells for 6 h, and then cellular lysates were used to capture AnxA6 by IP, from which the enriched AnxA6 was detected through western blot. (**E**) AnxA6 was decreased under ML-792 inhibitor exposure in HepG2 cells. HepG2 cells were treated with 10 μM ML-792 inhibitor for 48 h, and followed to perform immunofluorescence assay. The protein expression of AnxA6 was analyzed using the Pixel Intensity Spatial Correlation Analysis of software Image J. E-cadherin was taken as a positive control for cell membrane expression. Scale bars, 20 μm. Nuclei were counterstained with DAPI (blue). (**F**) The level of AnxA6 ubiquitination was detected in TPA-induced HEK293T cells under co-transfection of plasmids pFlag-AnxA6, pHA-ubiquitin and pMyc-SUMO1. (**G**) AnxA6 SUMOylation was reduced in TPA-induced EMT cells. The endogenous AnxA6 protein in TPA-induced cells was enriched from cell lysate by IP, in which the SUMOylated AnxA6 level was analyzed through western blot using SUMO1 antibody (up). The quantitative analysis of the SUMOylated AnxA6 levels in TPA induced-LO2 and HepG2 cells were also shown. The bar chart represented the ratio of SUMOylated AnxA6 to the total AnxA6 protein in the IP elution solution (bottom). *P < 0.05. (**H**) Cellular endogenous SUMO1 was enriched by IP to detect SUMOylated AnxA6 in TPA-induced HepG2 cells. (**I**) The native SUMO1-modified AnxA6 level was detected in MHCC-97 L and MHCC-97 H HCC cells with low/high metastasis abilities. Cellular endogenous AnxA6 was enriched by IP to detect SUMOylated AnxA6 against anti-SUMO1 antibody (up). The bar chart represents the ratio of SUMOylated AnxA6 *versus* the total AnxA6 protein in the IP elution solution (bottom). **P < 0.01

Increasing numbers of SUMOylated proteins have been confirmed to link with ubiquitination and tumorigenesis [30], and SUMOylation along with deSUMOylation is dynamic to regulate cell activities [31]. Moreover, our recent study has demonstrated that AnxA6 can be SUMOylated with SUMO1 at K156, K299 and K314 sites [16], but the biological function of AnxA6 SUMOylation in HCC is unknown. In this study, we have first confirmed that AnxA6 is modified by SUMO1 in HCC. Ubc9 acts as SUMO binding enzyme that catalyzes the target protein SUMOylation. We co-transfected plasmids pMyc-SUMO1 and pHA-Ubc9 in HEK293T cells to strengthen AnxA6 SUMOylation level, and detected SUMO1-AnxA6 conjugation from IP-enriched AnxA6 protein (Fig. 4A, Supplementary Fig. 3A).

Similarly, cell endogenous SUMO1-tagging AnxA6 was visible by IP enrichment against either AnxA6 (Fig. 4B, Supplementary Fig. 3B) or SUMO1 antibody (Fig. 4C, Supplementary Fig. 3C) in HEK293T cells. Compared with the native protein position of AnxA6 around 72 KDa, the endogenous SUMOylated AnxA6 was visible along with multiple slower migration bands with molecular weight bigger than 72 KDa (∼ 72 KDa for the endogenous AnxA6, ∼ 15 KDa for SUMO1), which showed AnxA6 was conjugated with SUMO1 (∼ 87 KDa).

SUMOylation has emerged as a key PTM that regulates protein stability and many biological processes [2]. Due to the antagonism effect between ubiquitination and SUMOylation, next we focused on the interplaying regulation of ubiquitination and SUMOylation within AnxA6 protein. We detected whether SUMOylation alters AnxA6 ubiquitination and ubiquitination-induced AnxA6 degradation. When the plasmid pHA-ubiquitin was co-transfected with pFlag-AnxA6 to cells for 48 h, the Flag-labeled AnxA6 protein was enriched. It was observed that AnxA6 was ubiquitinated by western blot analysis (Fig. 4D and Supplementary Fig. 3D, Lane 3). Meanwhile, AnxA6 ubiquitination was markedly inhibited after pMyc-SUMO1 transfection (Fig. 4D and Supplementary Fig. 3D, Lane 4), which indicated that SUMOylation would maintain AnxA6 protein stability from avoiding its ubiquitination.

ML-792, a chemical SUMOylation inhibitor, can reduce the AnxA6 SUMOylation [16]. We found that the expression of AnxA6 protein was decreased in cell membrane after ML-792 treatment (Pearson’s coefficient = 0.75, compared to untreated HepG2 cells) (Fig. 4E). It was further demonstrated that SUMOylation could stabilize the expression level of AnxA6 protein. Taken together, these data support the SUMOylation of AnxA6 is antagonistic with its protein ubiquitination, and SUMOylation stabilizes AnxA6 itself to retard protein degradation via ubiquitination.

### AnxA6 SUMOylation level is decreased in EMT-featured cells

Due to a lower expression of AnxA6 protein in TPA-induced EMT cells (Fig. 3C), we overexpressed AnxA6, SUMO1 and ubiquitin in wild-type and TPA-induced EMT cells to compare the ubiquitination levels of AnxA6 proteins. The result showed that the level of AnxA6 ubiquitination was higher in TPA-induced EMT cells (Fig. 4F, lane 3). Next, we compared the native AnxA6 SUMOylation degree in TPA-induced EMT cells and untreated parent cells. After the IP experiment, we adjusted the loading amount of the enriched AnxA6 protein to the same quantity in the following experiment. Under the same content of AnxA6 protein, the SUMOylated AnxA6 level was decreased in cells with EMT phenotype (Fig. 4G, Supplementary Fig. 3F). Conversely, we enriched the cellular exogenous SUMO1 using immunoprecipitation against SUMO1 antibody. The AnxA6 protein was detectable in the enriched proteins by western blot. Under the same content of SUMO1-conjugated protein, the AnxA6 level was decreased in TPA-induced HepG2 cells (Fig. 4H, Supplementary Fig. 3E).

Next, we measured the native AnxA6 SUMOylation degree in MHCC-97 L with low metastasis and MHCC-97 H cells with high metastasis. MHCC-97 H and MHCC-97 L cell lines are derived from the same parent HCC line, while MHCC-97 H has higher metastasis efficiency than MHCC-97 L. We enriched cellular endogenous AnxA6 by IP, and then detected SUMOylated AnxA6 against anti-SUMO1 antibody. Under the same quantity of AnxA6 in elute protein from immunoprecipitated beads, the SUMOylated AnxA6 level was lower in MHCC-97 H cells compared with MHCC-97 L cells (Fig. 4I, Supplementary Fig. 3G). These results demonstrate that AnxA6 SUMOylation level becomes weaker in HCC cells with high migration ability compared with the low migration of HCC cells.

### K579 is a major SUMOylation site of AnxA6 in HCC cells

We further identified the AnxA6 SUMOylation site(s) through MS/MS analysis and biological mutation validation to explore its biological function. Protein SUMOylation typically occurs at Lys (K) residues located within the consensus sequence ψKXE/D. Among the amino acid residues K75, K306, K418 and K579 of AnxA6 with a consensus SUMOylation sequence of ψKXE/D, the K579 was predicted to rank the highest possibility to be the potential SUMOylation site through bioinformatics analysis using the software SUMOsp (http://sumosp.biocuckoo.org/online.php) [32] (Supplementary Fig. 4A).

**Fig. 5:**
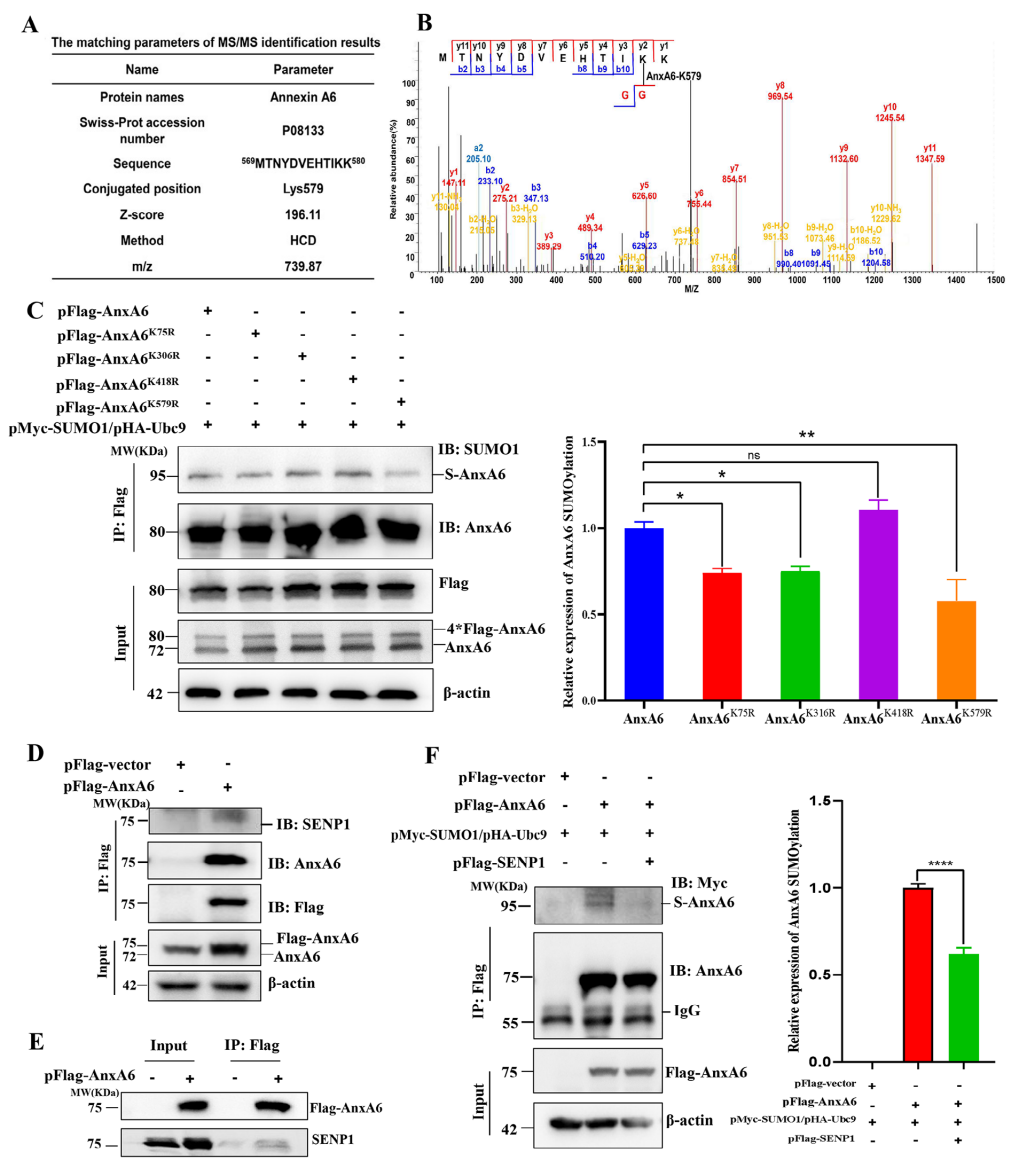
AnxA6 SUMOylation is identified to conjugate with SUMO1 at K579 residue, and SENP1 cooperates with AnxA6 to perform deSUMOylation of AnxA6 in HCC cells. (**A**-**B**) K579 site of the peptide ^569^MTNYDVEHTIKK^580^ from AnxA6 was identified to SUMOylate by MS/MS analysis in HCD fragmentation mode. (**C**) The K579 site mutation reduced AnxA6 SUMOylation level in HepG2 cells. pFlag-AnxA6^K75R^, pFlag-AnxA6^K306R^, pFlag-AnxA6^K418R^ and pFlag-AnxA6^K579R^ were AnxA6 site mutated plasmids, each with a single K point mutation, to validate SUMOylation level changes of AnxA6 by western blot (left). Quantification of the SUMOylated AnxA6 levels in HepG2 cells (right). The bar chart represented the ratio of SUMOylated Flag-tag AnxA6 to the total AnxA6 protein in the IP elution solution. ns, no statistical; *P < 0.05; **P < 0.01; ***P < 0.001. (**D**-**E**) SENP1 cooperated with AnxA6 to perform AnxA6 deSUMOylation. HepG2 cells were transfected plasmids pFlag-AnxA6 with or without pFlag-SENP1 for 48 h to detect by IP using the anti-Flag antibody. (**F**) SENP1 overexpression decreased the SUMOylation of AnxA6 in HepG2 cells. HepG2 cells were transfected pFlag-AnxA6 with or without pFlag-SENP1 for 48 h to detect by IP using the anti-Flag antibody (left). Quantification of the SUMOylated AnxA6 levels in HepG2 cells transfected pFlag-AnxA6 with or without pFlag-SENP1(right). The bar chart represented the ratio of SUMOylated Flag-tag AnxA6 to the total AnxA6 protein in the IP elution solution. ****P < 0.0001. S-AnxA6: Flag-tagging SUMOylated AnxA6, IP: immunoprecipitation, IB: immunoblot, Input: same account of cell lysate to load

The pFlag-AnxA6 and pHis-SUMO1^T95K^ (T95 mutation to K) plasmids were simultaneously co-transfected into HEK293T cells to identify the AnxA6 SUMOylation site by MS/MS analysis. The matching parameters of MS/MS identification were summarized in Fig. 5A. Using the mutant SUMO tagging method, the target K579-containing peptide ^569^MTNYDVEHTIKK^580^ of AnxA6 was identified as the modified residue by MS/MS via HCD scan (Fig. 5B).

Then we further constructed site mutation plasmids, including pFlag-AnxA6^K75R^, pFlag-AnxA6^K306R^, pFlag-AnxA6^K418R^ and pFlag-AnxA6^K579R^, through site-directed mutagenesis to verify the SUMOylation. The genes encoding mutant amino acid residues were confirmed by DNA sequencing (Supplementary Fig. 4B). The wild-type plasmid pFlag-AnxA6 and 4 mutant plasmids were respectively co-transfected with plasmids pHA-Ubc9 and pMyc-SUMO1 into HepG2 cells to observe influence on AnxA6 SUMOylation. The Flag-AnxA6-associated proteins were pulled down by IP with anti-Flag antibody in HepG2 cells. Among the 4 substitutions, the single site mutation of K579R led to a greatly decreased SUMOylation level of AnxA6 (Fig. 5C, Lane 5) compared with the wild-type AnxA6 in HepG2 cells (Fig. 5C, Lane 1). The most obvious SUMO-tagging AnxA6 bands with a molecular weight of 95 KDa (the expected normal size of 4*Flag-AnxA6 is 80 kDa). A similar result was provided in Supplementary Fig. 4C. It was noted that several SUMO1-modified AnxA6 bands appeared under a long exposure since SUMO1 can rely on its lysine to form poly-chains to conjugate with AnxA6.

Our recent study showed that a single site mutation of K299R greatly impaired AnxA6 SUMOylation level in HEK293T and epithelial cancer cells ^[16]^. Therefore, we compared the effects of site mutation of K299R and K579R on AnxA6 SUMOylation in HCC cells. It was worth mentioning that K299 was not located in the consensus motif ψKxD/E. Mutations in both K299R and K579R resulted in significantly decreased SUMOylation level of AnxA6 (Supplementary Fig. 4D, Lane 2 and Lane 3) compared with the wild-type AnxA6 in HepG2 cells. Based on the findings, we conclude that K579 is the major SUMOylation site of AnxA6 that is located within the consensus SUMO-motif sequence.

### SENP1 cooperates with AnxA6 to confer deSUMOylation of AnxA6

SUMOylation with deSUMOylation is a reversible and dynamic process that maintains balance to play critical roles in many physiological activities. DeSUMOylation is usually regulated by a SUMO-specific protease SENP1 targeting SUMO1-modified substrate proteins. Therefore, we tested whether SENP1 interacted with AnxA6 to confer AnxA6 deSUMOylation. The plasmid pFlag-AnxA6 was transfected into HepG2 cells for 48 h to enrich the AnxA6-binding protein complex to detect SENP1 level. The result showed that SENP1 (∼ 75 KDa) interacts with AnxA6 in HepG2 cells (Fig. 5D-E). When SENP1 was co-expressed with AnxA6 in HepG2 cells, the level of SUMOylated AnxA6 was reduced (Fig. 5F). These results support SENP1 acts as the deSUMOylation enzyme to perform SUMO1 removal from AnxA6.

### AnxA6 deSUMOylation promotes cell migration by disassociating the binding of AnxA6 with RHOU

**Fig. 6:**
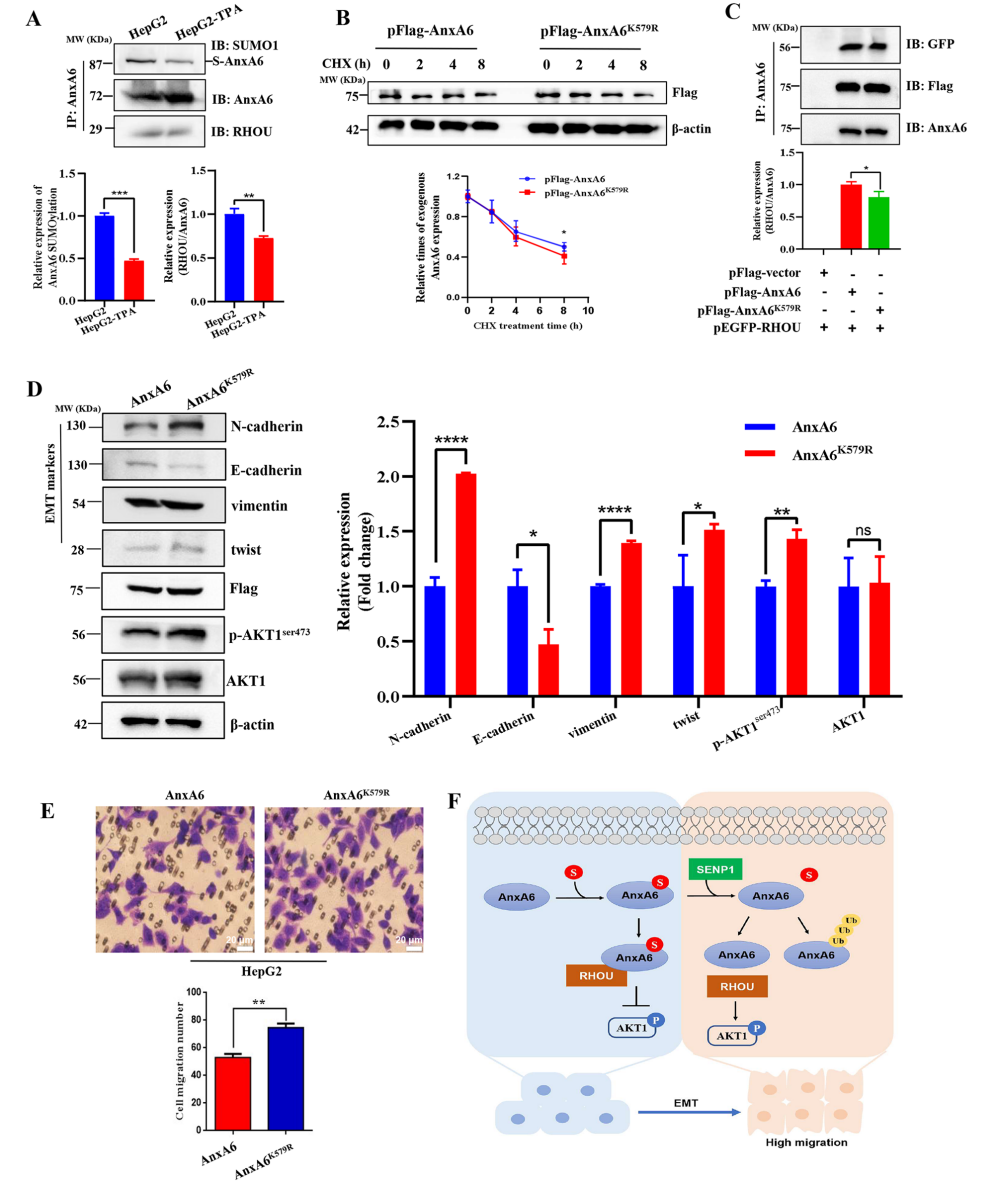
AnxA6 deSUMOylation promotes cell migration. (**A**) The binding of RHOU with AnxA6 became weakened in TPA-induced HepG2 cells. The cell lysates of wild-type HepG2 and TPA-induced HepG2 cells were performed IP to capture the AnxA6-binding complex, detected by western blot analysis with SUMO1, AnxA6 and RHOU antibodies. S-AnxA6: Flag-tagging SUMOylated AnxA6, IP: immunoprecipitation. (**B**) The half-life of AnxA6 was prolonged than that of AnxA6^K579R^. The plasmids pFlag-AnxA6 or pFlag-AnxA6^K579R^ were respectively transfected into HepG2 cells, then incubated with 100 μg/mL CHX for 0, 2, 4 and 8 h. The cell lysates were used to detect indicated protein (up). AnxA6 levels were quantified from three independent experiments and represented as the mean ± SD (bottom). (**C**) Compared to wild-type AnxA6, the binding of the K579R mutant to RHOU was weaker in HepG2 cells. Co-transfection of plasmids pEGFP-RHOU, pHis-SUMO1, pFlag-AnxA6 or pFlag-AnxA6^K579R^ in HepG2 cells was to enrich Flag-tagging AnxA6 by IP, and then EGFP-tagging RHOU expression was detected in IP precipitation. S-AnxA6: Flag-tagging SUMOylated AnxA6, IP: immunoprecipitation, IB: immunoblot, Input: same cell lysate to load. (**D**) DeSUMOylation attenuates AnxA6 role in suppressing the EMT process. The plasmids pFlag-AnxA6 or pFlag-AnxA6^K579R^ were respectively transfected into HepG2 cells to measure EMT markers and p-AKT1^ser473^ levels (left). Quantitative results were represented as the mean ± SD of three separate experiments (right). β-actin was used as an internal control. ns, no statistical; *P < 0.05; **P < 0.01; ***P < 0.001. (**E**) DeSUMOylation of AnxA6 enhanced cell migration. The plasmids pFlag-AnxA6 or pFlag-AnxA6^K579R^ were respectively transfected into HepG2 cells and performed transwell assays. (**F**) A mechanistic model of AnxA6 deSUMOylation contributes to cell migration and HCC progression. AnxA6 inhibits the cell migration ability of HCC that depends on its SUMOylation level. However, the SUMOylation-mediated protective mechanism is disrupted by a deSUMOylase SENP1, which leads to deSUMOylation and degradation of AnxA6 protein in EMT cells, and promotes HCC progression through RHOU/AKT1 signaling

The interplay of SUMOylation and deSUMOylation of AnxA6 with intracellular signaling proteins plays key roles in cell migration and HCC progression. To confirm whether AnxA6 SUMOylation affects the binding of AnxA6 with RHOU, we enriched cellular endogenous AnxA6 in TPA-induced HepG2 cells by IP, and then detected RHOU level. When the level of AnxA6 SUMOylation was decreased, the binding of RHOU with AnxA6 became weakened (Fig. 6A, Supplementary Fig. 5A).

Meanwhile, we investigated the influence of K579-mediated SUMOylation of AnxA6 on protein function and cancer cell phenotype. Plasmid pFlag-AnxA6 or pFlag-AnxA6^K579R^ was transfected to HepG2 cells to measure the half-life of the wild-type Flag-tagging AnxA6 and mutant protein of AnxA6^K579R^. The half-life of the AnxA6 protein was dramatically lengthened compared with the mutant AnxA6^K579R^ (Fig. 6B). This result indicates SUMOylation stabilizes AnxA6 to counteract ubiquitin-mediated degradation.

Next, we detected the binding of RHOU with AnxA6 in HepG2 cells under transfection of pFlag-AnxA6 or site mutant pFlag-AnxA6^K579R^. Under the same quantity of Flag-tagging AnxA6 or AnxA6^K579R^, the binding of mutation AnxA6^K579R^ with EGFP-RHOU was weakened (Fig. 6C, Lane 3) compared to the wild type of AnxA6 (Fig. 6C, Lane 2). Moreover, the mutant AnxA6^K579R^ greatly weakened its inhibition ability on the EMT process compared with wild-type AnxA6, resulting in an increase of p-AKT1^ser473^, N-cadherin and twist, a decrease of E-cadherin (Fig. 6D, Supplementary Fig. 5B). It was consistent that cell migration ability was increased in the AnxA6^K579R^-expressing cells compared with the AnxA6-overexpressing HepG2 cells (Fig. 6E, *p* < 0.05).

Combined with the data of Figs. 2 and 6, these results demonstrate that AnxA6 deSUMOylation leads to disassociation of the binding of AnxA6 with RHOU, then the free form of RHOU and p-AKT1^ser473^ are upregulated to facilitate EMT process and cell migration (Fig. 6F).

### AnxA6 level negatively correlates with SENP1 expression in HCC

**Fig. 7:**
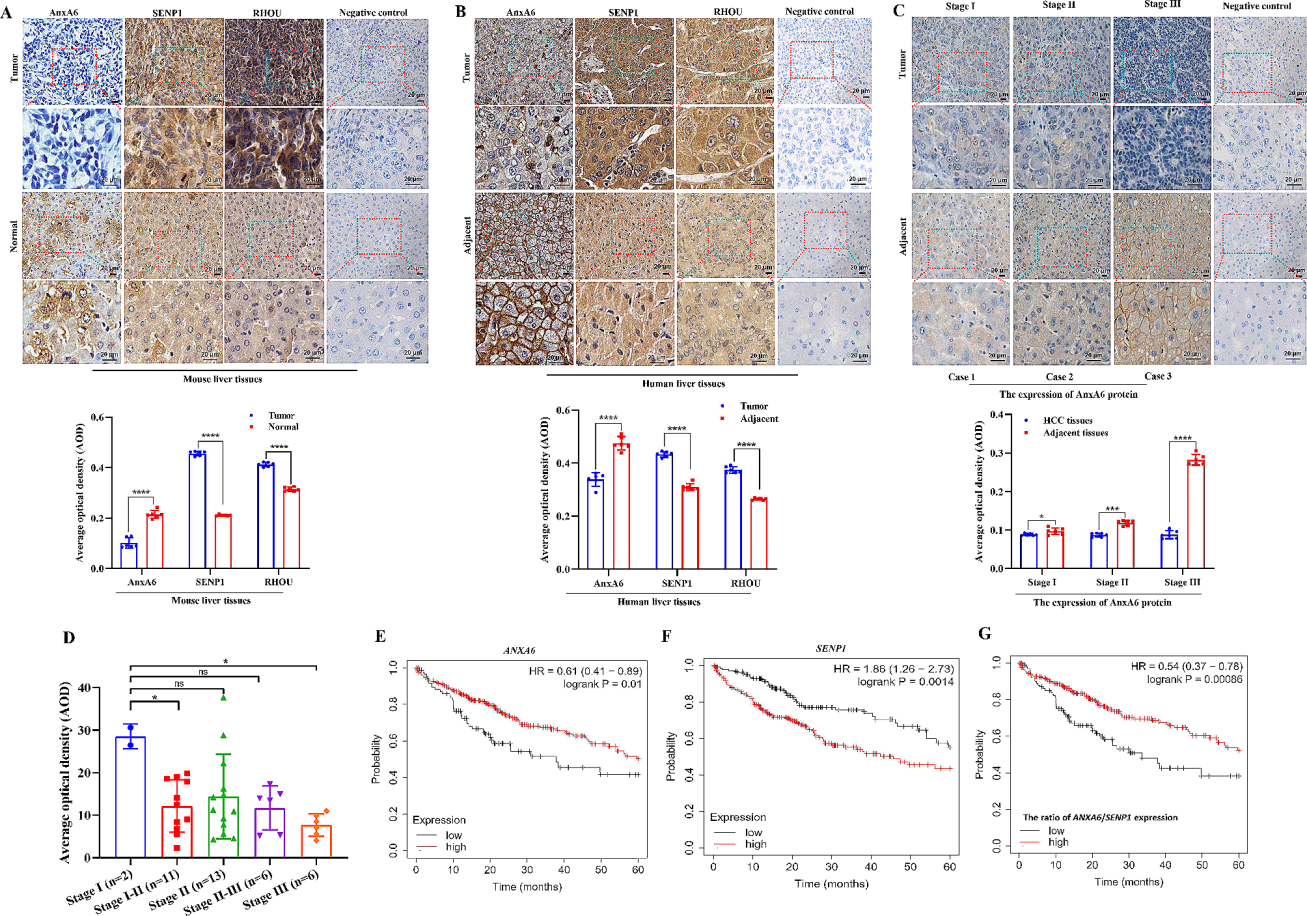
AnxA6 level negatively correlates with SENP1 expression in HCC tissues. (**A**) Representative immunohistochemical staining and quantitative results of AnxA6, RHOU and SENP1 in mouse orthotopic tumor tissues and normal liver tissues (*n* = 3, left). Stained slides without the primary antibody were used as a negative control for IHC staining in HCC tissues. At least 6 randomly selected fields in the examined liver sections were measured in each group by the software Image Fiji (right). Scale bars, 20 μm. *P < 0.05; **P < 0.01; ***P < 0.001. (**B**) Representative immunohistochemical staining and quantitative results of AnxA6, RHOU and SENP1 in human HCC tissues and adjacent tissues (*n* = 3, left). Stained slides without the primary antibody were used as a negative control for IHC staining in HCC tissues. At least 6 randomly selected fields in the examined liver sections were measured in each group by software Image Fiji (right). Scale bar, 20 μm. *P < 0.05; **P < 0.01; ***P < 0.001. (**C**) AnxA6 downregulation in HCC tissues (*n* = 15) with different pathologic stages was validated by IHC (left). Quantitative results of AnxA6 in HCC tissues with different pathologic stages (right). *P < 0.05; **P < 0.01; ***P < 0.001. Scale bar, 20 μm. (**D**) The expression of AnxA6 in HCC tissues (*n* = 38) with different pathologic stages. *P < 0.05. ns, no statistical. (**E**) Low expression of *ANXA6* in HCC was related to poor overall survival using Kaplan-Meier plotter database analysis. (**F**) High expression of *SENP1* in HCC indicated poor overall survival. (**G**) The higher expression ratio of *ANXA6*/*SENP1* indicated the higher survival rate of HCC patients. HR: hazard ratio. *p*-values were calculated using the log-rank test

AnxA6 protein was mainly expressed on cell membrane in HCC tissues (Supplementary Fig. 5D). Subsequently, we analyzed the correlation between the deSUMOylase SENP1, AnxA6 and RHOU in HCC tissues. Firstly, we detected the expression level of RHOU, AnxA6 and SENP1 protein in mouse orthotopic hepatoma tissues. The murine HCC tissues injected with Hepa1-6 cells in vivo are highly similar to human HCC in histology, which is widely used to construct a mouse orthotopic hepatoma model [33]. Three cases of mouse orthotopic hepatoma tissues were used for IHC experiments. Compared with mouse normal liver tissues, the IHC staining revealed that the levels of SENP1 and RHOU were a relatively high expression in mouse liver cancer tissues, but AnxA6 was decreased. AnxA6 downregulation is associated with high expression levels of SENP1 and RHOU in mouse orthotopic HCC tissues (Fig. 7A).

Next, we detected the expression level of AnxA6, RHOU and SENP1 protein in human HCC tissues collected from surgically resected cases. We performed IHC analysis in 3 randomly selected HCC tissues and patients’ autologous adjacent tissues. Compared with the corresponding adjacent non-cancer tissues, the level of SENP1 and RHOU was upregulation in HCC tissues, but AnxA6 level was downregulation (Fig. 7B). These results indicate the reverse expression association of SENP1 and AnxA6 in HCC tissues, which is consistent with the conclusion in mouse orthotopic hepatoma.

To further validate the correlation between AnxA6 expression profiling and tumor grade, we performed IHC analysis in 15 HCC tissues with different tumor stages and patients’ autologous adjacent tissues. The detailed clinicopathological characteristics of HCC samples were displayed in Supplementary Table 1. As a result, AnxA6 protein showed downregulation in HCC tissues with average scores of 3.33 ± 1.35 compared to the counterparts (*p* < 0.05) (Table 1). Among these cases, 13 HCC tissues (86.7%) showed a low level of AnxA6 with average scores 2.92 ± 0.86, and 2 cases (13.3%) showed a high level with average scores 6. Notably, AnxA6 protein was obviously decreased in HCC tissues with pathologic stages III (Fig. 7C). Moreover, we used a commercial tissue array with 38 HCC tissues to measure the protein expression of AnxA6 (Supplementary Table 2). The results showed that AnxA6 protein was obviously reduced in HCC tissues with pathologic stage I-II and stage III compared to tissues of the pathologic stage I (*p* < 0.05) (Fig. 7D, Supplementary Fig. 5C). These results support that AnxA6 downregulation expression is closely related to the high degree of HCC malignancy.

**Table 1:** AnxA6 expression profiling between 15 pairs of HCC and adjacent tissues

Immuno-reactivity	HCC tissues (*n* = 15)			Adjacent tissues (*n* = 15)		*p* value
	Percentage	Average score		Percentage	Average score	
Total	100% (15/15)	3.33 ± 1.35		100%(15/15)	5.60 ± 2.44	*p* < 0.01
Low (+)	86.7% (13/15)	2.92 ± 0.86		33.3% (5/15)	2.60 ± 0.89	
High (++)	13.3% (2/15)	6		66.7% (10/15)	7.10 ± 1.20	

The immune-reactivity differences between HCC tissues and Adjacent tissues groups were estimated using Student’s t-test. *p* < 0.01 was considered statistically significant

Percentage: (specific cases/total cases)

Low AnxA6 level (+) was scored 1–4, while the high level (++) was more than 4 scores

We also evaluated the correlation between *ANXA6* gene expression and clinical outcomes using the Kaplan-Meier Plotter analysis. HCC patients with high expression of *ANXA6* had a longer overall survival compared with those with low expression of *ANXA6* gene (Hazard ratio [HR] = 0.61, *p* = 0.01) (Fig. 7E). Conversely, Low expression of *ANXA6* is related with poor overall survival for HCC patients.

We further analyzed the expression relevance of the *ANXA6* gene and *SENP1* in bioinformatics using the online HCC database. Compared with normal liver tissues, the *SENP1* gene was increased in the HCC of TCGA-LIHC dataset from GEPIA database (Supplementary Fig. 5E), and the negative expression correlation between *ANXA6* and *SENP1* was obvious in HCC samples from TCGA-LIHC dataset (Supplementary Fig. 5F-G). Moreover, through Kaplan Meier Plotter database analysis, HCC patients with low *SENP1* expression had a significantly higher survival rate than those patients with a high *SENP1* expression (HR = 1.86, *p* = 0.0014) (Fig. 7F).

We further verified whether the expression ratio of *ANXA6* versus *SENP1* (*ANXA6*/*SENP1*) would have a more favorable prognostic performance. The Kaplan-Meier plot assay showed that a higher ratio of *ANXA6*/*SENP1* in HCC tissues indicated a higher survival rate of HCC patients (HR = 0.54, *p* = 0.00086) (Fig. 7G). These results support that SENP1 acts as deSUMOylation enzyme of AnxA6 to negatively link with AnxA6 expression, and the combination of *ANXA6* and *SENP1* (ratio of *ANXA6*/*SENP1*) would be a potential prognosis biomarker for HCC overall survival.

## Discussion

As a member of the annexin family, AnxA6 role in cancer development is rather complicated, it acts as a promoting factor in some cancers and is also considered to exert tumor-suppressing activities [34, 35]. AnxA6 represents almost 0.25% of the total protein mass in the liver [9]. Moreover, the expression of AnxA6 is the basis to ensure the recovery and regeneration of the liver after transplantation or liver surgery [36]. Our study has confirmed that AnxA6 level is decreased in HCC cells and tissues, which is consistent with previous literature results [37]. In addition, we have further proven that AnxA6 overexpression inhibits tumorigenesis in the subcutaneous xenograft HCC model and orthotopic hepatoma model.

On the other hand, we have revealed that AnxA6 is decreased in EMT-featured HCC cells by TPA inducement [38] and HCC tissues with higher pathologic stages. The EMT process is thought to be a key event in intrahepatic dissemination and distal metastasis of HCC cells [39]. Herein, we further investigated the deSUMOylation-driven mechanism of AnxA6 downregulation in EMT-featured HCC cells. SUMOylation is one of the PTMs that ensure optimized cellular processes, including regulating signaling pathways, cell survival, and stress adaptation to maintain a balanced homeostatic state. Abnormal PTMs are associated with cellular dysfunction and the occurrence of cancer [40]. There is growing evidence that epigenetic silencing of AnxA6 is a common mechanism in cancers. For instance, the CpG-rich AnxA6 promoter is heavily methylated in EGFR-overexpressing cancer cells with low AnxA6 levels [41]. Similarly, AnxA6 mRNA is down-regulated through promoter methylation in gastric cancer [12]. In our study, the results are the first time to reveal that deSUMOylation of AnxA6 is associated with its degradation *via* the ubiquitin-proteasome pathway in EMT-featured HCC cells.

Protein SUMOylation is a dynamic regulation in physiologic conditions, and the imbalance of SUMOylation and deSUMOylation is associated with the occurrence of cancer [42]. SUMO1 is involved in normal physiological functions of liver cells. SUMO1 modification maintains a variety of biological processes such as signal transduction, transcription regulation, nuclear and cytoplasmic transport, protein interaction and other functions in liver cells [43]. The approaches for SUMOylation identification mainly include bioinformatics combined with amino acid site-directed mutagenesis and MS-based proteomics analysis [2, 44]. Herein, AnxA6 is identified as one potential SUMO1 substrate by using a peptide-level immunoprecipitation enrichment-MS strategy [45] and amino acid site-directed mutagenesis strategy [46]. It is worth mentioning that we have identified multiple SUMOylation sites of AnxA6 by MS. In the current study, we have confirmed that AnxA6 is major modified by SUMO1 at K579 residue which plays a key function in HCC cell migration and tumor growth.

The substrate protein undergoes SUMOylation at different lysine residues, which greatly expands the biological function of the substrate protein. For example, SUMO E3 ligase Trim38 SUMOylated cGAS at K231 and K479 residues during the early phase of viral infection, preventing the polyubiquitination and protein degradation of cGAS [47]. However, the SUMO protein was conjugated onto lysine residues K335, K372 and K382 of cGAS, which suppressed its DNA-binding, oligomerization and nucleotidyltransferase activities [48]. The biological function of SUMOylation of AnxA6 at different residues requires further investigation.

Like many scaffolding proteins, annexins regulate protein-protein interactions [8]. AnxA6 is involved in mediating cell migration [26, 49]. Due to the interplay effect among ubiquitination, SUMOylation and deSUMOylation, SUMOylation is helpful for AnxA6 to be more stable to prevent its degradation *via* protein ubiquitination. In addition, our studies have found that AnxA6 SUMOylation confers its binding with RHOU to inhibit cell migration *via* suppressing the RHOU/AKT1 signaling pathway. Conversely, deSUMOylation of AnxA6 under SENP1, a deSUMOylated enzyme, facilitates RHOU dissociating with AnxA6 to activate RHOU/p-AKT1^ser473^-involved cell migration and EMT process.

AnxA6 overexpression also inhibits EGFR signaling [26] and H-Ras/MAPK signaling [41]. And EGFR and Ras are upstream proteins of RHOU [24]. Furthermore, both EGFR and H-Ras have been linked to EMT in HCC [50, 51]. Our data provided that AnxA6 inhibits cell migration via reducing RHOU/Akt activity (Fig. 2). However, the interactions between SUMOylated AnxA6 and EGFR, and effects on downstream cell migration need to be clarified in further studies.

We have first time investigated AnxA6 and SENP1 expression with HCC clinical outcome relevance. In HCC tissues, SENP1 protein is usually high, which is reversely linked with the AnxA6 downregulation level. Moreover, the combined detection of AnxA6 and SENP1 shows a better clinical prognosis value for HCC overall survival than any single molecule. The Kaplan‑Meier analysis supports higher survival rates for HCC patients with higher gene expression ratios of *ANXA6*/*SENP1*. The interaction of multiple genes and factors is an important cause of tumor occurrence and development. When studying the influence of target genes on survival prognosis, a comprehensive analysis of the combination of two or multiple genes/proteins for the prognosis is feasible and more precise compared to a single molecule. For example, the combination of *RARbeta* with either *DAPK* or *RASSF1A* showed a significantly shorter overall survival of those malignant mesothelioma patients who had both genes methylated compared to those with only one or no epigenetic alteration [52]. The combination of PGRMC1 and ATP1A1 protein levels can serve as a promising indicator of the prognosis of renal cell carcinoma [53]. The increased co-expression of PD-L1 and TIM3 is associated with poor overall survival of esophageal squamous cell carcinoma patients [54]. Our results support the idea that the prognostic value of a combination of two or muti-genes is superior to the impact of an individual gene alone on the overall survival of cancer patients.

## Conclusions

AnxA6 downregulation in HCC is associated with the downregulated SUMOylation level of AnxA6, and the SUMO1 modification of AnxA6 at the residue K579 stabilizes AnxA6 protein itself to counteract ubiquitin-mediated degradation. AnxA6 deSUMOylation leads to disassociate the binding of AnxA6 with RHOU, subsequently the free RHOU and p-AKT1 are upregulated to facilitate cell migration and EMT progression. Moreover, SENP1 is negatively correlated with AnxA6 protein expression, and a higher gene expression ratio of *ANXA6/SENP1* indicates a poor overall survival of HCC patients.

### Electronic supplementary material

Below is the link to the electronic supplementary material.

Supplementary Material 1

Supplementary Material 2

Supplementary Material 3

Supplementary Material 4

## Data Availability

No datasets were generated or analysed during the current study.

## Abbreviations

AnxA6: Annexin A6
EMT: Epithelial-mesenchymal transition
HCC: Hepatocellular carcinoma
PTMs: Post-translational modifications
SENP: Sentrin-specific protease
SUMO: Small ubiquitin-like modifier
TPA: 12-O-tetradecanoylphorbol-13-acetate
Ubc9: SUMO-conjugating enzyme 9
