# Supplementary material for: SUMOylation of annexin A6 retards cell migration and tumor growth by suppressing RHOU/AKT1–involved EMT in hepatocellular carcinoma
Source: Cell Commun Signal. 2024 Apr 2;22:206. doi: 10.1186/s12964-024-01573-2 (PMC10986105; doi:10.1186/s12964-024-01573-2)
Supplement: Supplementary file 1 — Supplementary Material 1 [file 12964_2024_1573_MOESM1_ESM.docx]

**Supplementary Table S1. The detailed information of 15 pairs human hepatocellular cancer tissues.**

| **Patients** | **Type of tissues** | **Primary cancer** | **Metastases** | **Gender** | **Age** | **Pathological stage** |
| --- | --- | --- | --- | --- | --- | --- |
| 1 | HCC | Yes | No | Male | 61 | Ⅰ |
| 2 | HCC | Yes | No | Male | 63 | Ⅰ |
| 3 | HCC | Yes | No | Male | 64 | Ⅰ |
| 4 | HCC | Yes | No | Male | 64 | Ⅱ |
| 5 | HCC | Yes | No | Male | 34 | Ⅱ |
| 6 | HCC | Yes | No | Male | 43 | Ⅱ |
| 7 | HCC | Yes | No | Male | 52 | Ⅱ |
| 8 | HCC | Yes | No | Female | 63 | Ⅱ |
| 9 | HCC | Yes | No | Female | 56 | Ⅱ |
| 10 | HCC | Yes | No | Male | 63 | Ⅱ |
| 11 | HCC | Yes | No | Male | 53 | Ⅱ |
| 12 | HCC | Yes | No | Male | 52 | Ⅱ |
| 13 | HCC | Yes | No | Male | 42 | Ⅲ |
| 14 | HCC | Yes | No | Female | 68 | Ⅲ |
| 15 | HCC | Yes | No | Male | 55 | Ⅲ |

HCC: Hepatocellular carcinoma.
