# Supplementary material for: SUMOylation of annexin A6 retards cell migration and tumor growth by suppressing RHOU/AKT1–involved EMT in hepatocellular carcinoma
Source: Cell Commun Signal. 2024 Apr 2;22:206. doi: 10.1186/s12964-024-01573-2 (PMC10986105; doi:10.1186/s12964-024-01573-2)
Supplement: Supplementary file 2 — Supplementary Material 2 [file 12964_2024_1573_MOESM2_ESM.docx]

**Supplementary Table S2. Detailed information of tissue array.**

**
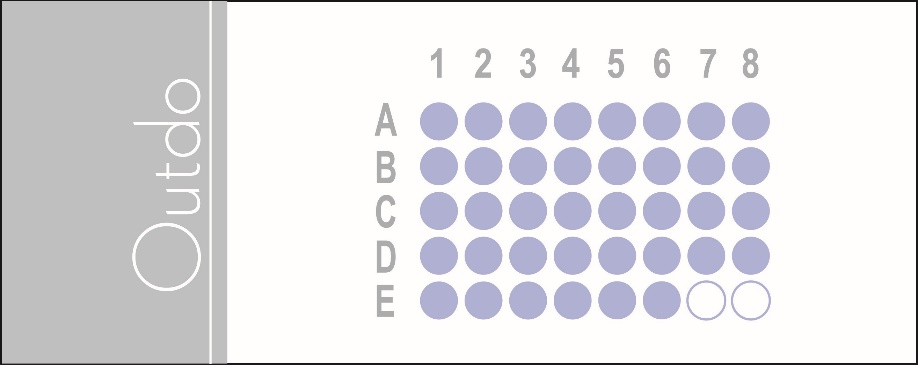
**

| **Position** | **Gender** | **Age** | **Pathological type** | **Pathological stage** |
| --- | --- | --- | --- | --- |
| (A,1) | male | 64 | Hepatocellular carcinoma | Ⅰ |
| (A,2) | female | 59 | Hepatocellular carcinoma | Ⅰ |
| (A,3) | male | 42 | Hepatocellular carcinoma | Ⅰ-Ⅱ |
| (A,4) | male | 48 | Hepatocellular carcinoma | Ⅰ-Ⅱ |
| (A,5) | male | 37 | Hepatocellular carcinoma | Ⅰ-Ⅱ |
| (A,6) | male | 56 | Hepatocellular carcinoma | Ⅰ-Ⅱ |
| (A,7) | male | 54 | Hepatocellular carcinoma | Ⅰ-Ⅱ |
| (A,8) | female | 57 | Hepatocellular carcinoma | Ⅰ-Ⅱ |
| (B,1) | male | 45 | Hepatocellular carcinoma | Ⅰ-Ⅱ |
| (B,2) | male | 43 | Hepatocellular carcinoma | Ⅰ-Ⅱ |
| (B,3) | male | 58 | Hepatocellular carcinoma | Ⅰ-Ⅱ |
| (B,4) | male | 61 | Hepatocellular carcinoma | Ⅰ-Ⅱ |
| (B,5) | male | 63 | Hepatocellular carcinoma | Ⅰ-Ⅱ |
| (B,6) | female | 19 | Hepatocellular carcinoma | Ⅱ |
| (B,7) | male | 39 | Hepatocellular carcinoma | Ⅱ |
| (B,8) | male | 77 | Hepatocellular carcinoma | Ⅱ |
| (C,1) | male | 71 | Hepatocellular carcinoma | Ⅱ |
| (C,2) | male | 53 | Hepatocellular carcinoma | Ⅱ |
| (C,3) | male | 72 | Hepatocellular carcinoma | Ⅱ |
| (C,4) | male | 50 | Hepatocellular carcinoma | Ⅱ |
| (C,5) | male | 48 | Hepatocellular carcinoma | Ⅱ |
| (C,6) | male | 52 | Hepatocellular carcinoma | Ⅱ |
| (C,7) | female | 63 | Hepatocellular carcinoma | Ⅱ |
| (C,8) | male | 50 | Hepatocellular carcinoma | Ⅱ |
| (D,1) | female | 58 | Hepatocellular carcinoma | Ⅱ |
| (D,2) | male | 39 | Hepatocellular carcinoma | Ⅱ |
| (D,3) | male | 41 | Hepatocellular carcinoma | Ⅱ-Ⅲ |
| (D,4) | female | 38 | Hepatocellular carcinoma | Ⅱ-Ⅲ |
| (D,5) | male | 51 | Hepatocellular carcinoma | Ⅱ-Ⅲ |
| (D,6) | male | 49 | Hepatocellular carcinoma | Ⅱ-Ⅲ |
| (D,7) | male | 32 | Hepatocellular carcinoma | Ⅱ-Ⅲ |
| (D,8) | male | 55 | Hepatocellular carcinoma | Ⅱ-Ⅲ |
| (E,1) | male | 31 | Hepatocellular carcinoma | Ⅲ |
| (E,2) | male | 60 | Hepatocellular carcinoma | Ⅲ |
| (E,3) | male | 40 | Hepatocellular carcinoma | Ⅲ |
| (E,4) | male | 38 | Hepatocellular carcinoma | Ⅲ |
| (E,5) | male | 36 | Hepatocellular carcinoma | Ⅲ |
| (E,6) | male | 71 | Hepatocellular carcinoma | Ⅲ |
