# Supplementary material for: SUMOylation of annexin A6 retards cell migration and tumor growth by suppressing RHOU/AKT1–involved EMT in hepatocellular carcinoma
Source: Cell Commun Signal. 2024 Apr 2;22:206. doi: 10.1186/s12964-024-01573-2 (PMC10986105; doi:10.1186/s12964-024-01573-2)
Supplement: Supplementary file 3 — Supplementary Material 3 [file 12964_2024_1573_MOESM3_ESM.docx]

**
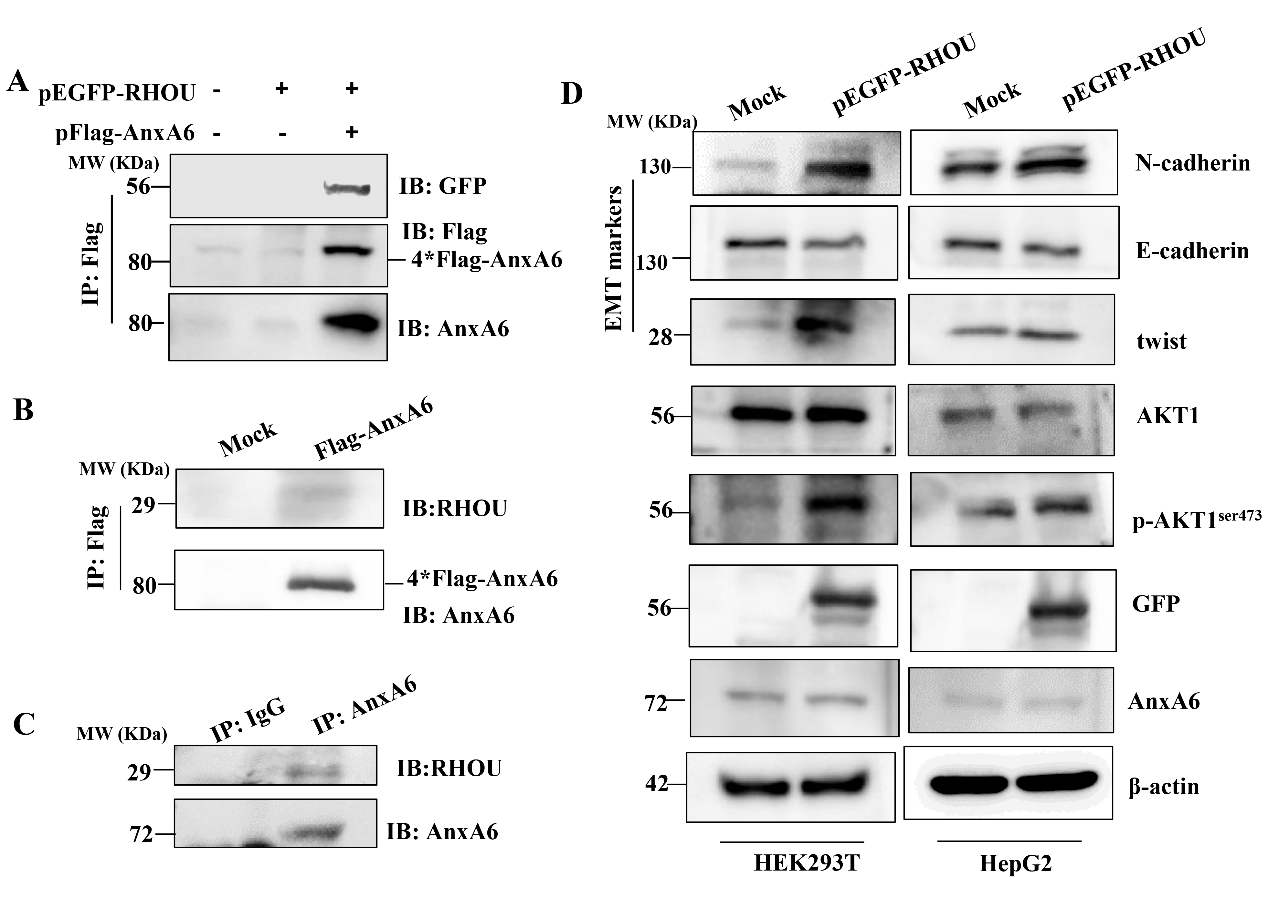
**

**Supplementary Figure 1.** (A) Ectopic expression of AnxA6 was confirmed to interact with GFP-tag RHOU protein. Co-transfection with plasmids pEGFP-RHOU and pFlag-AnxA6 into HepG2 cells, then enriched Flag-tagging AnxA6 by co-IP, and the AnxA6-interacting RHOU was detected in protein precipitation using anti-GFP antibody. (B) Ectopic expression of AnxA6 was confirmed to interact with endogenous RHOU protein. HepG2 cells were transfected with pFlag-AnxA6 plasmids for 48 h, the lysates were performed IP to capture AnxA6 and subsequently immunoblotted with RHOU antibody. (C) Endogenous AnxA6 interacts with RHOU. The lysates of HepG2 cells were performed IP to capture endogenous AnxA6 and subsequently immunoblotted with RHOU antibody. (D) Protein expression level of EMT markers and p-AKT1^ser473^ in HEK293T and HepG2 cells. HEK293T and HepG2 cells were transiently transfected with pEGFP-RHOU for 48h, then several key EMT markers and p-AKT1^ser473^ were detected.

**
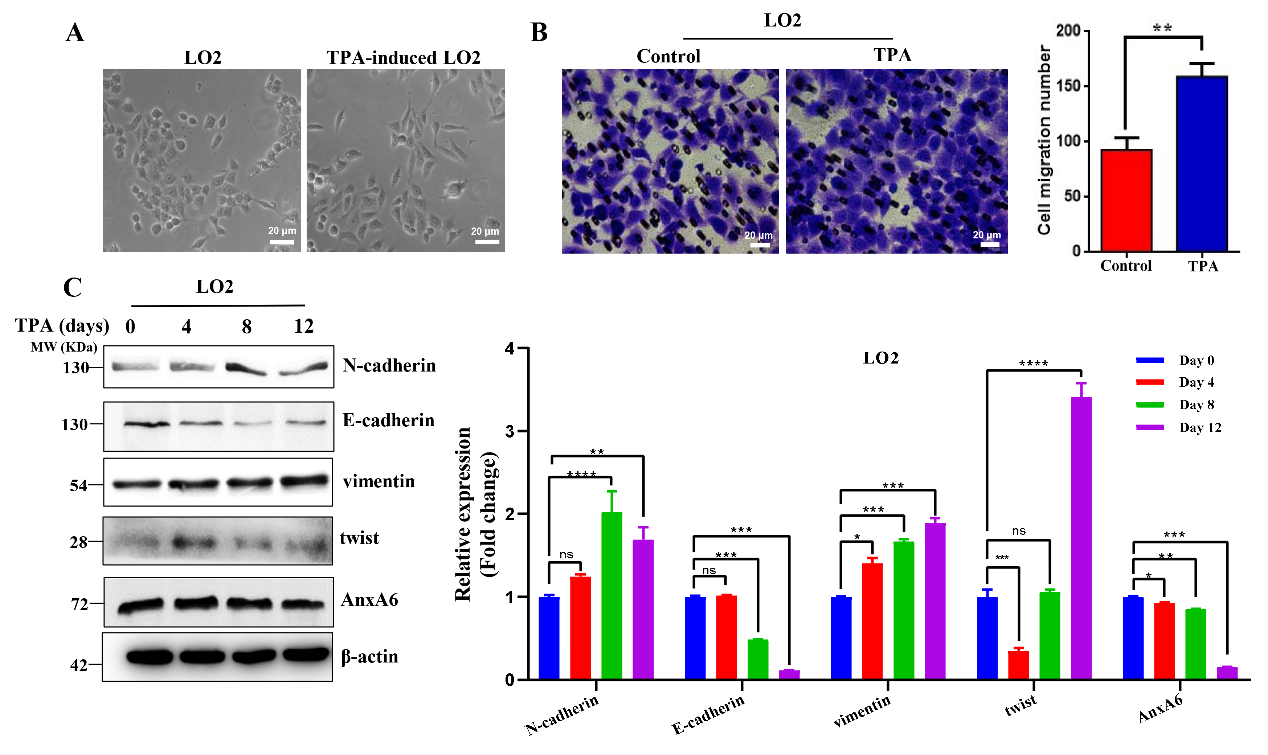
**

**Supplementary Figure 2.** (A) Cell morphology changes of LO2 cells under TPA exposure. LO2 cells were treated with 100 ng/mL TPA for 0, 4, 8, 12 days, and the images were captured with a microscope. Scale bars, 20 μm. (B) Cell migration ability was increased in TPA-induced EMT cells. Cell migration of the TPA-induced LO2 cells were analyzed using transwell assays. Images were captured with a microscope, and three random fields were selected to count the number of migration cells. *p<0.05, **p<0.01. Scale bars, 20 μm. (C) EMT biomarkers were dynamically expressed in TPA-induced cells at different treatment days. LO2 cells were treated with 100 ng/mL TPA for 0, 4, 8, 12d, and cell proteins were collected for western blot with indicated antibodies (left). Quantification of protein levels of EMT markers in TPA-induced LO2 cells. Data were represented as the mean±SD of three separate experiments (right). ns, no statistical; *P<0.05; **P<0.01; ***P<0.001.

**
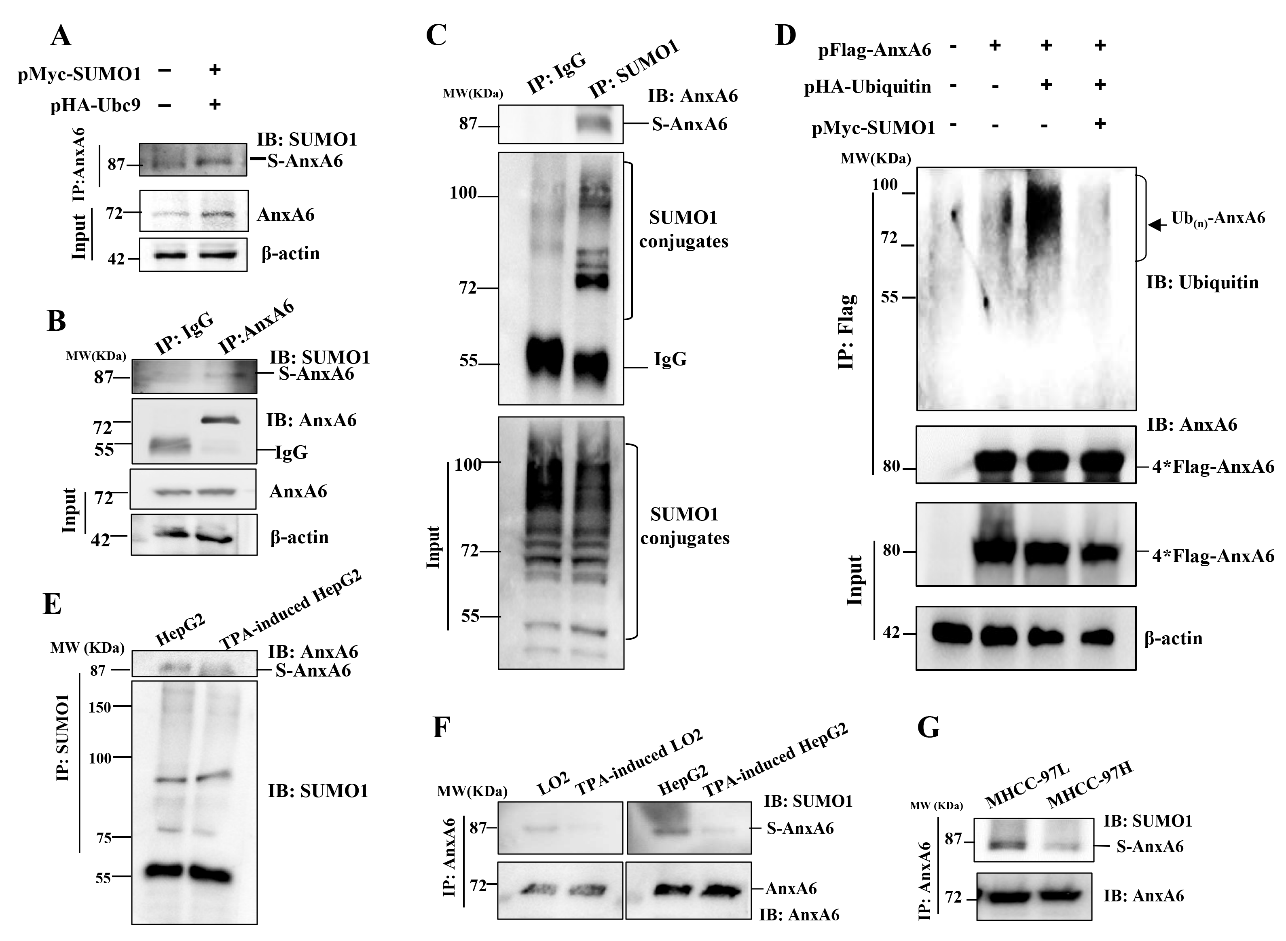
**

**Supplementary Figure 3.** (A) The SUMO1-modified AnxA6 was detected in HEK293T cells under co-transfection of plasmids pHA-Ubc9 and pMyc-SUMO1. The SUMOylated AnxA6 was captured by IP with anti-AnxA6. (B-C) The endogenous SUMOylated AnxA6 was measured in HEK293T cells. The SUMOylated AnxA6 was captured by IP with anti-AnxA6 (B) or anti-SUMO1 (C) antibody, then respectively immunoblotted with anti-SUMO1 or anti-AnxA6 antibody. The normal IgG was a nonspecific binding control for IP. S-AnxA6: Flag-tagging SUMOylated AnxA6. (D) Antagonism between SUMOylation and ubiquitination of AnxA6 protein. After the target plasmids were transfected into HepG2 cells for 48 h, 20 μM MG132 as a proteasome inhibitor was added to incubate with cells for 6 h, then cellular lysates were used to capture AnxA6 by IP, from which the enriched AnxA6 was detected through western blot. (E) Cellular endogenous SUMO1 was enriched by IP to detect SUMOylated AnxA6 in TPA-induced HepG2 cells. (F) AnxA6 SUMOylation was reduced in TPA-induced EMT cells. The endogenous AnxA6 protein in TPA-induced cells was enriched from cell lysate by IP, in which the SUMOylated AnxA6 level was analyzed through western blot using SUMO1 antibody. (G) The native SUMO1-modified AnxA6 level was detected in MHCC-97L and MHCC-97H HCC cells with low/high metastasis abilities. Cellular endogenous AnxA6 was enriched by IP to detect SUMOylated AnxA6 against anti-SUMO1 antibody.

**
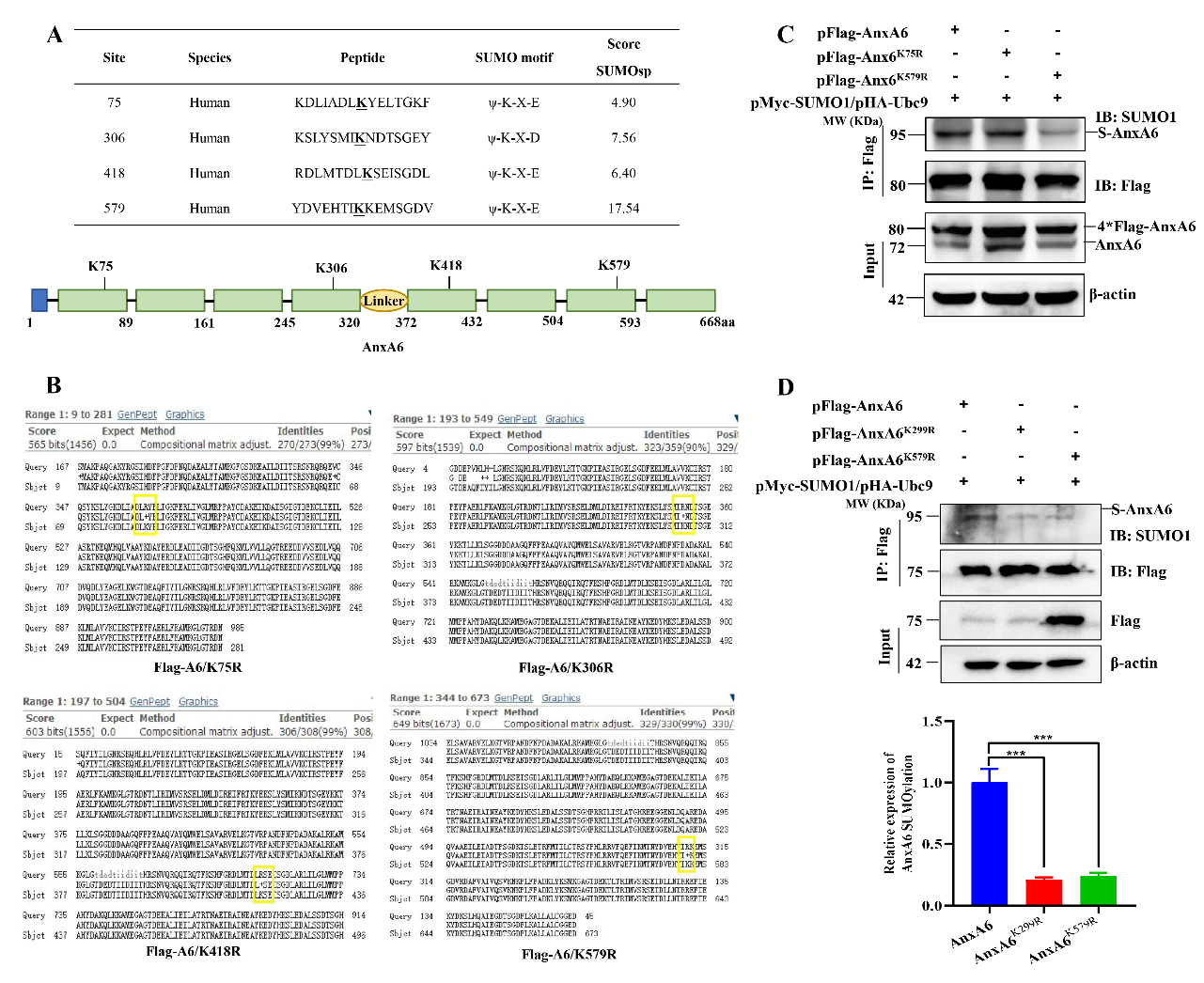
**

**Supplementary Figure 4.** (A) The K579 was predicted to rank the highest possibility to be the potential SUMOylation site through bioinformatics analysis using the software SUMOsp. (B) DNA Sequencing for mutant plasmids. The plasmids pFlag-AnxA6^K75R^, pFlag-AnxA6^K306R^, pFlag-AnxA6^K418R^and pFlag-AnxA6^K579R^ were constructed through site-directed mutagenesis. And the sequencing results were compared in NCBI's Nucleotide Blast. (C-D) Flag-AnxA6, its SUMOylated site mutants, HA-Ubc9 and Myc-SUMO1 were expressed in HepG2 cells. Then, the cells were lysed and the proteins analyzed by IP using the anti-Flag antibody followed by Western blotting. pFlag-AnxA6^K75R^, pFlag-AnxA6^K306R^, pFlag-AnxA6^K418R^ and pFlag-AnxA6^K579R^ were AnxA6 site mutated plasmids, each with a single K point mutation, to validate SUMOylation level changes of AnxA6 by Western blot. S-AnxA6: Flag-tagging SUMOylated AnxA6, IP: immunoprecipitation, IB: immunoblot, Input: same account of cell lysate to load.


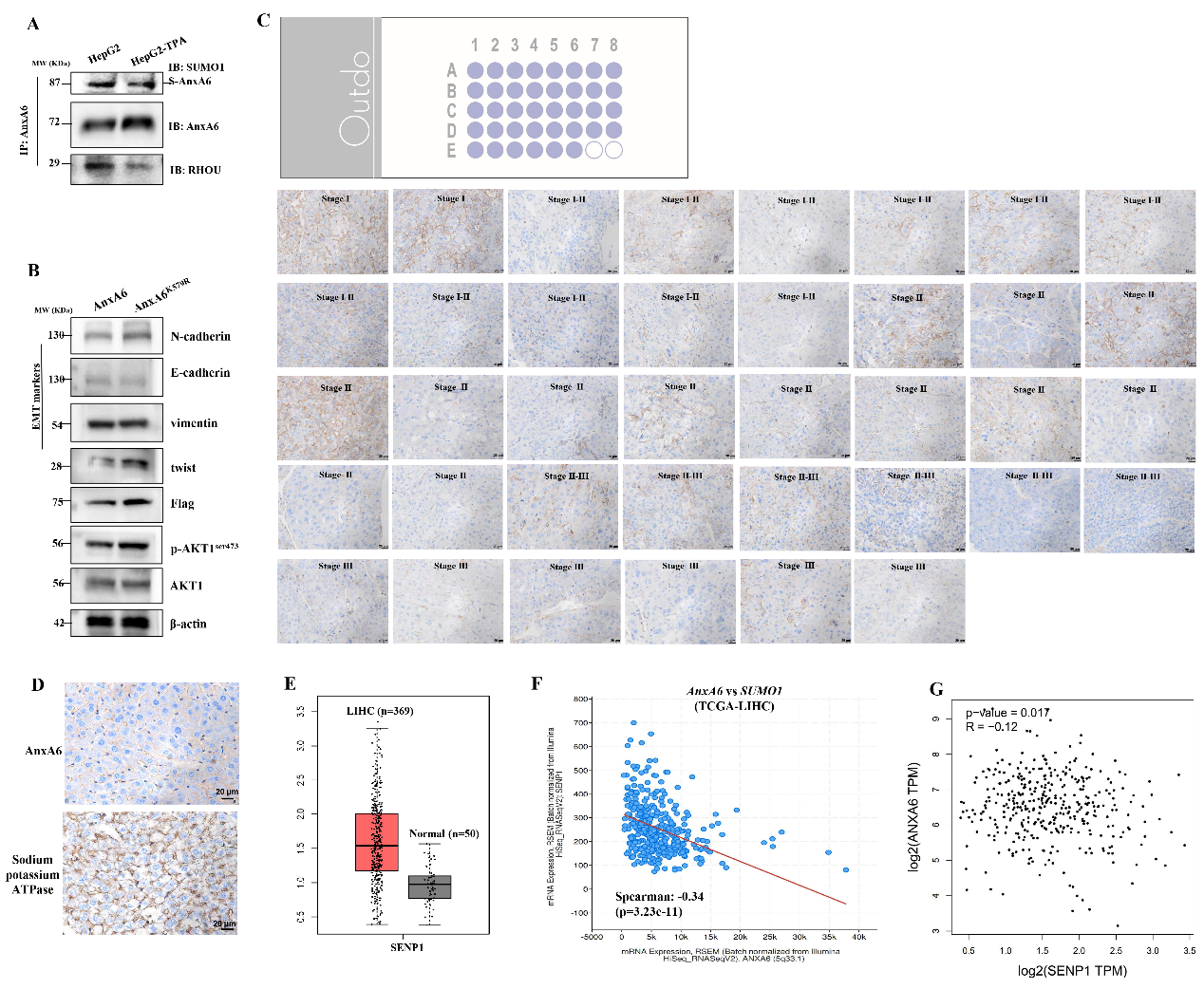


**Supplementary Figure 5.** (A) The binding of RHOU with AnxA6 became weakened in TPA-induced HepG2 cells. The cell lysates of wild type HepG2 and TPA-induced HepG2 cells were performed IP to capture AnxA6-binding complex, detected by western blot analysis with SUMO1, AnxA6 and RHOU antibodies. S-AnxA6: Flag-tagging SUMOylated AnxA6, IP: immunoprecipitation. (B) DeSUMOylation attenuates AnxA6 role of suppressing the EMT process. The plasmids pFlag-AnxA6 or pFlag-AnxA6^K579R^ was respectively transfected into HepG2 cells to measure EMT markers and p-AKT1^ser473^ levels. (C) Representative immunohistochemical staining of AnxA6 in HCC tissue array (n=38) with different pathologic stages. Scale bars, 20 μm. (D) The distribution of AnxA6 and membrane protein Sodium potassium ATPase in HCC tissues. Scale bars, 20 μm. (E) *SENP1* gene level is increased in tumors from TCGA-LIHC dataset of GEPIA. LIHC, Liver hepatocellular carcinoma. GEPIA, Gene expression profiling interactive analysis. Red: tumor samples. Black: normal samples. (F-G) *ANXA6* negatively correlates with *SENP1* expression in LIHC from TCGA dataset in GEPIA and cBioPortal database.
