# Supplementary material for: SUMOylation of annexin A6 retards cell migration and tumor growth by suppressing RHOU/AKT1–involved EMT in hepatocellular carcinoma
Source: Cell Commun Signal. 2024 Apr 2;22:206. doi: 10.1186/s12964-024-01573-2 (PMC10986105; doi:10.1186/s12964-024-01573-2)
Supplement: Supplementary file 4 — Supplementary Material 4 [file 12964_2024_1573_MOESM4_ESM.pdf]

Figure 1A

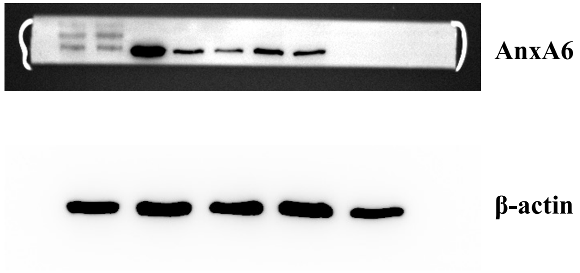

Figure 1B

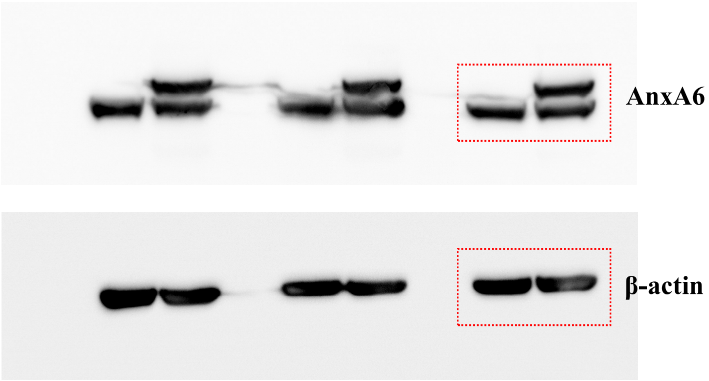

Figure 1C

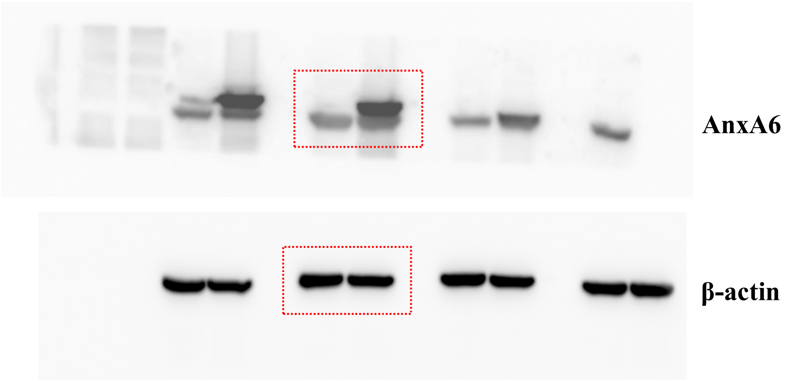

Figure 2A

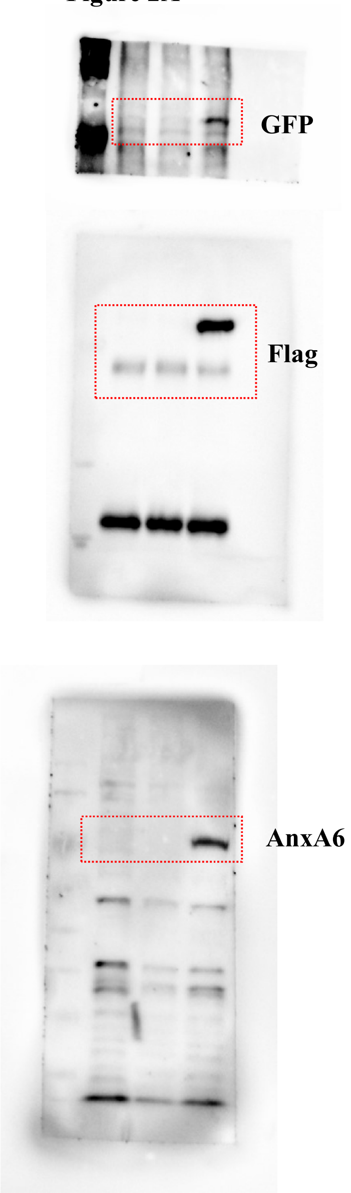

Figure 2B

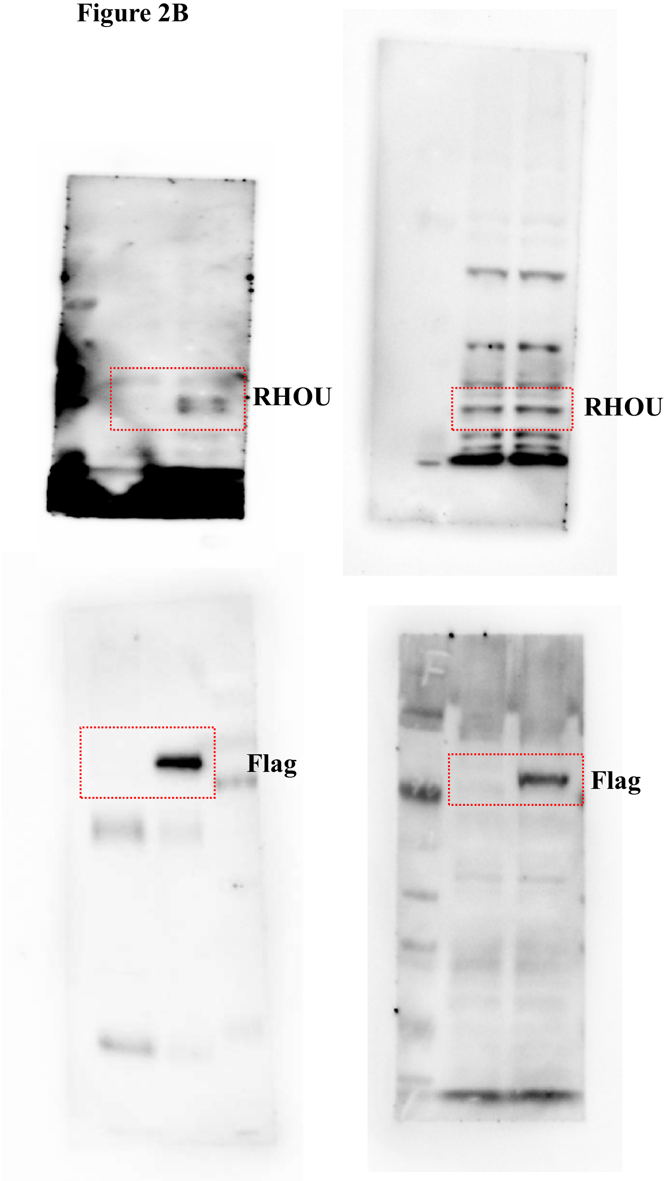

Figure 2C

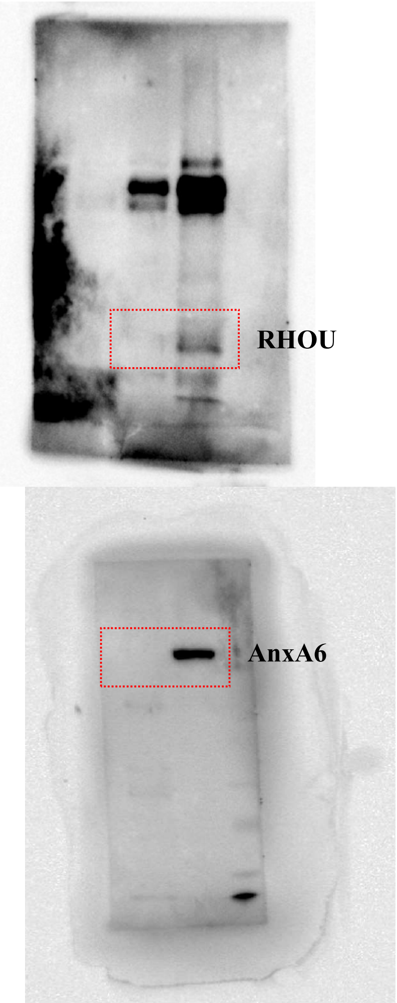

Figure 2D

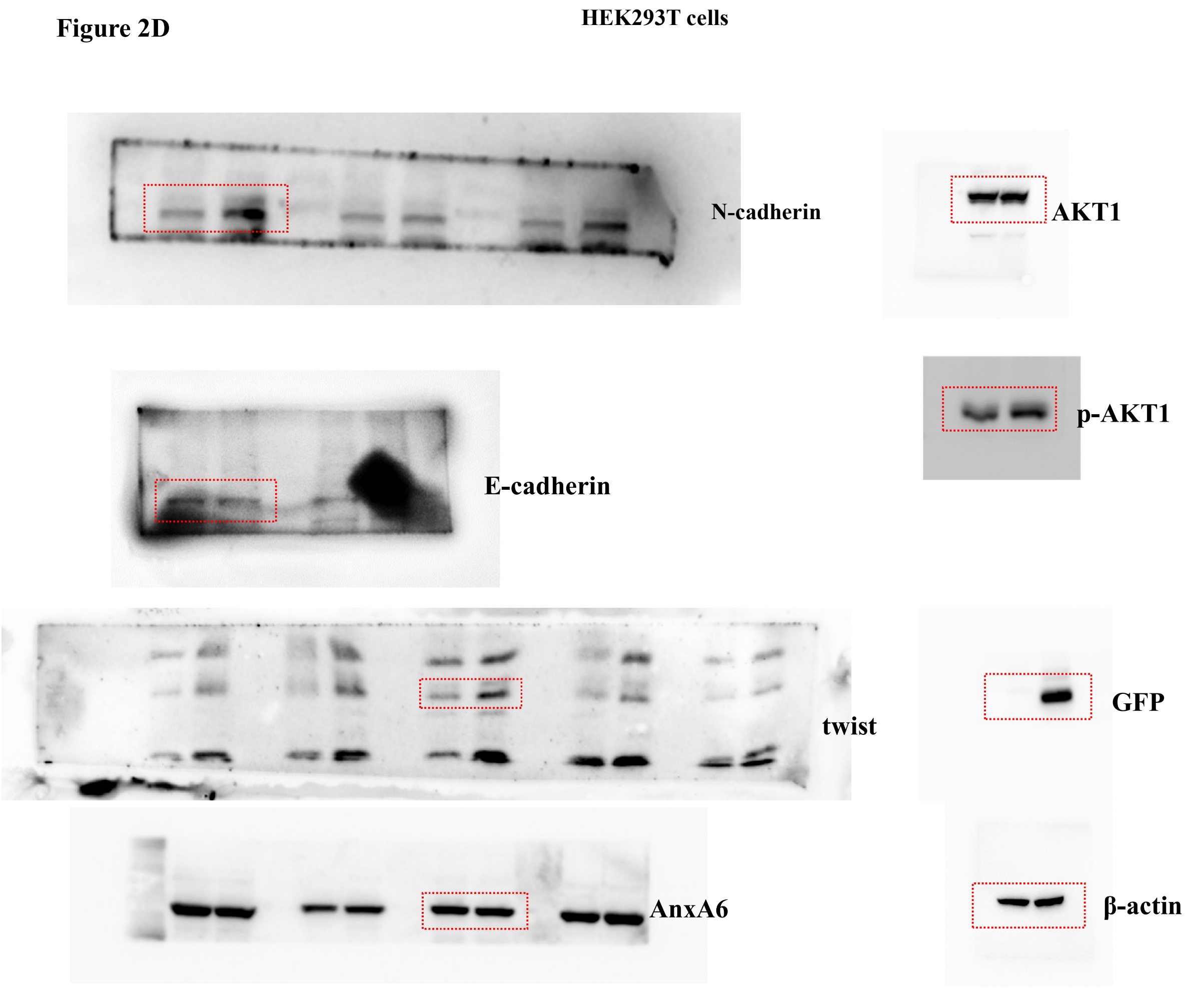
HEK293T cells
N-cadherin
AKT1
p-AKT1
E-cadherin
GFP
twist
AnxA6
β-actin

Figure 2D HepG2 cells

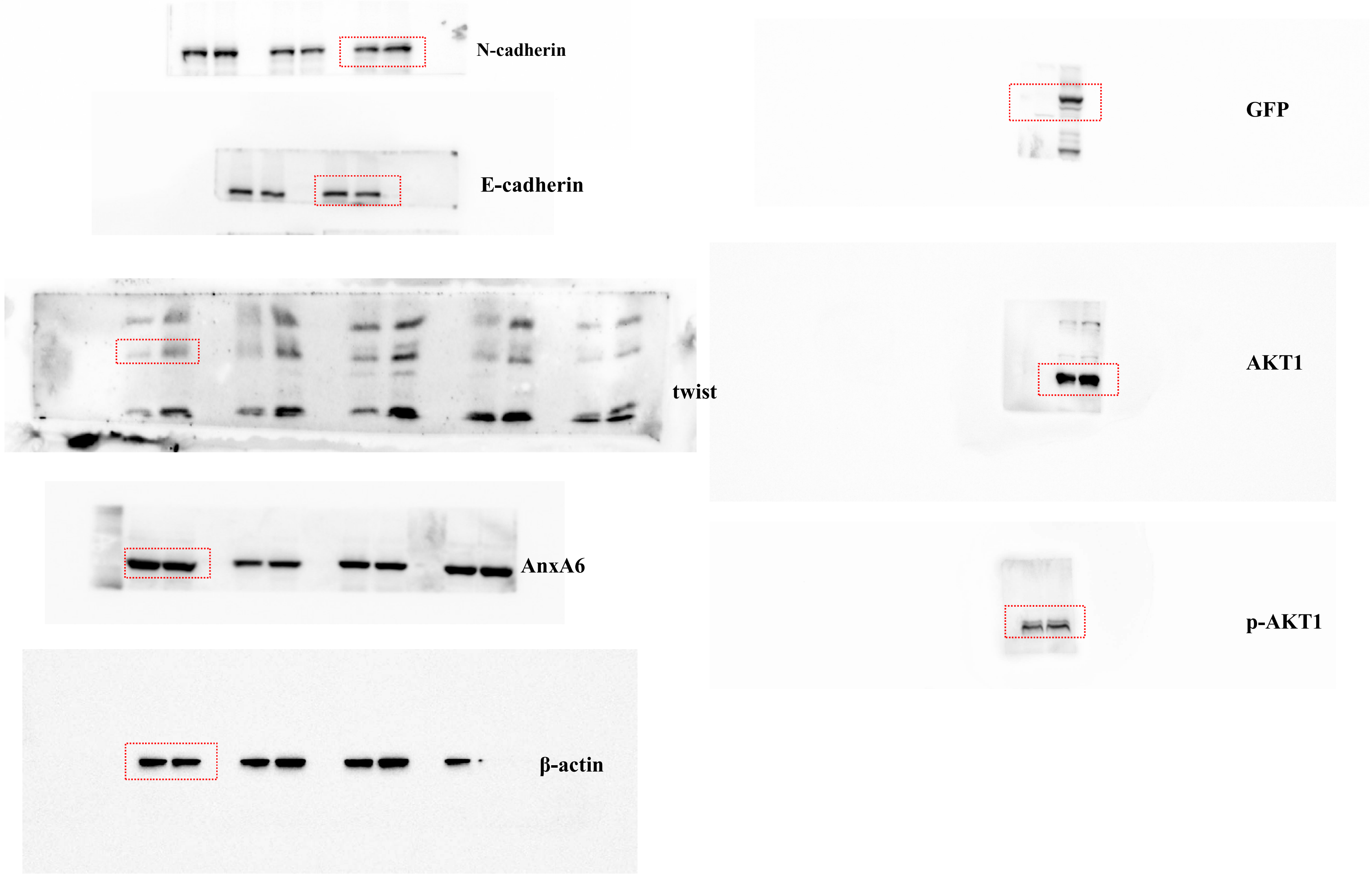

Figure 2E

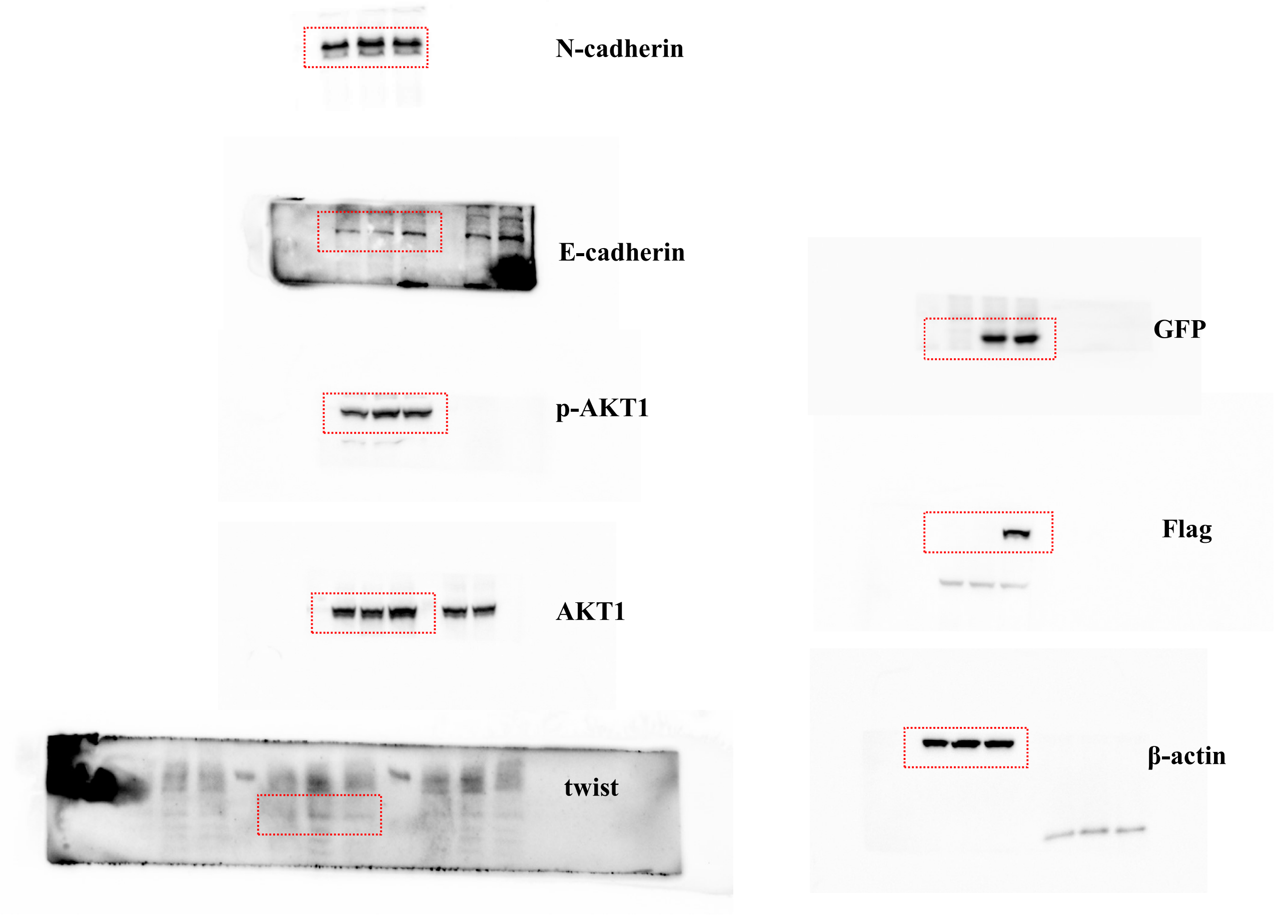

Figure 2F

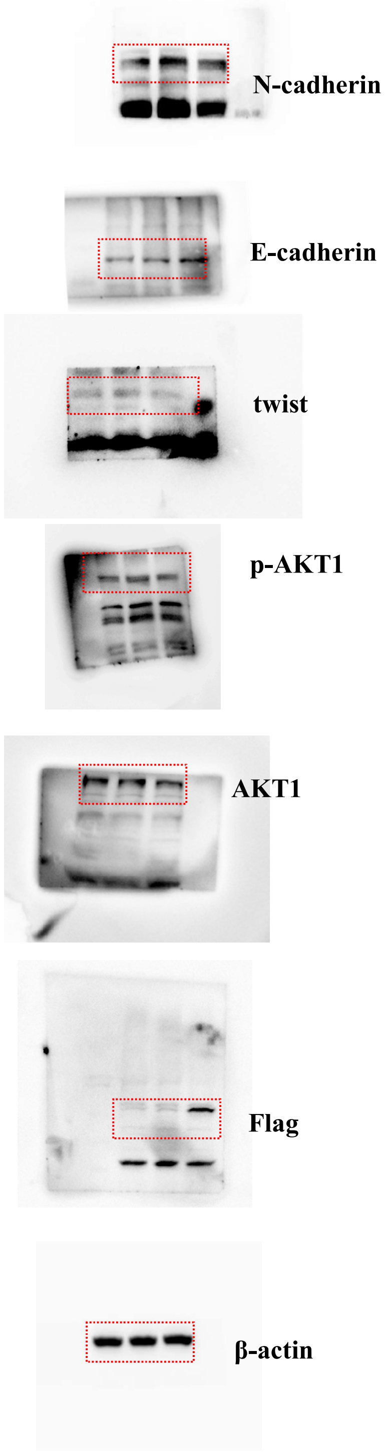

Figure 2H

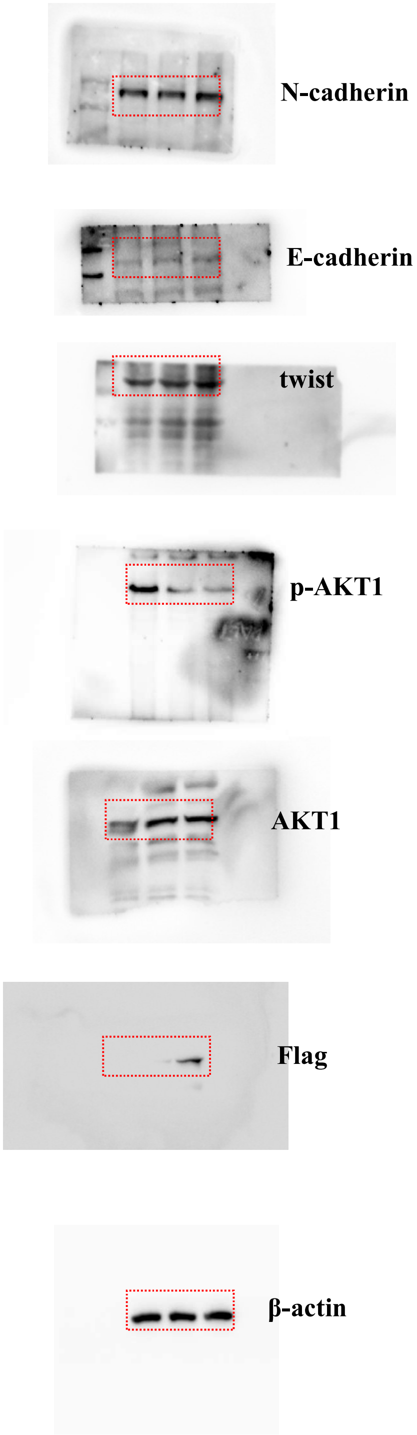

Figure 3C

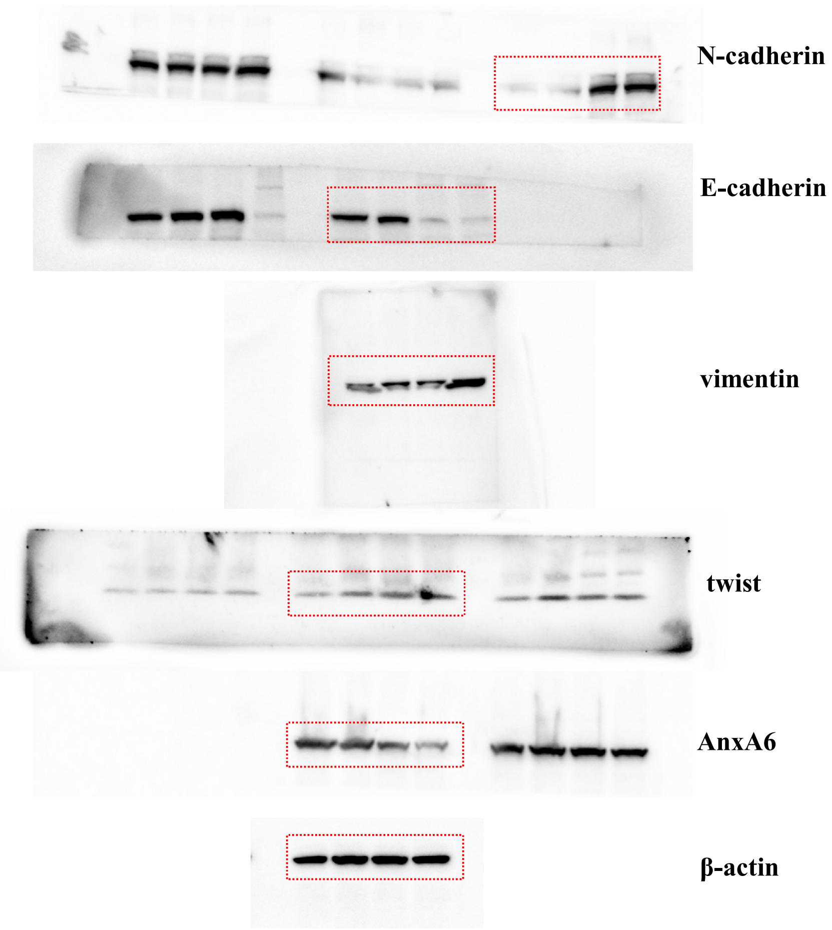

Figure 3D

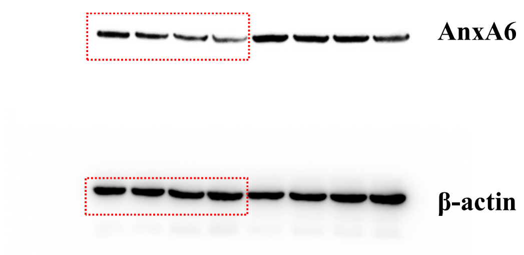

Figure 3E

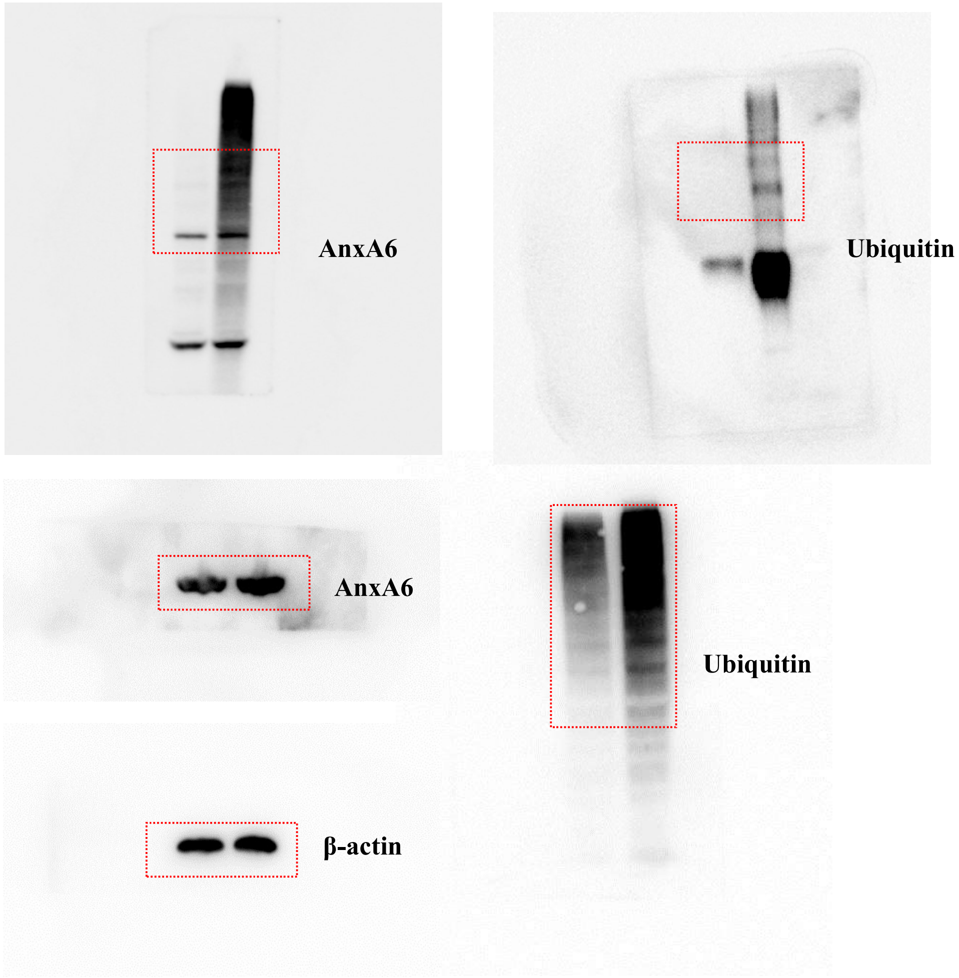

Figure 3F

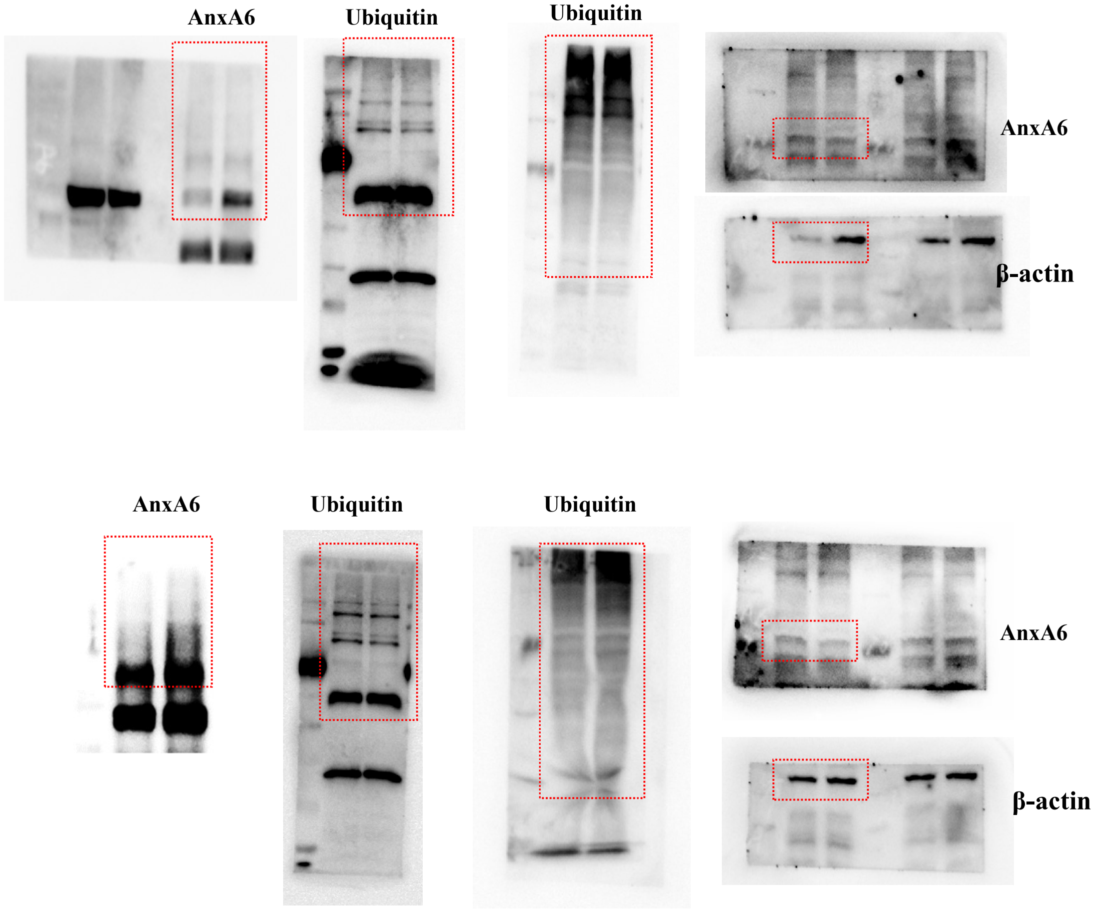

AnxA6
Ubiquitin
Ubiquitin
AnxA6
β-actin
AnxA6
Ubiquitin
Ubiquitin
AnxA6
β-actin

Figure 4A

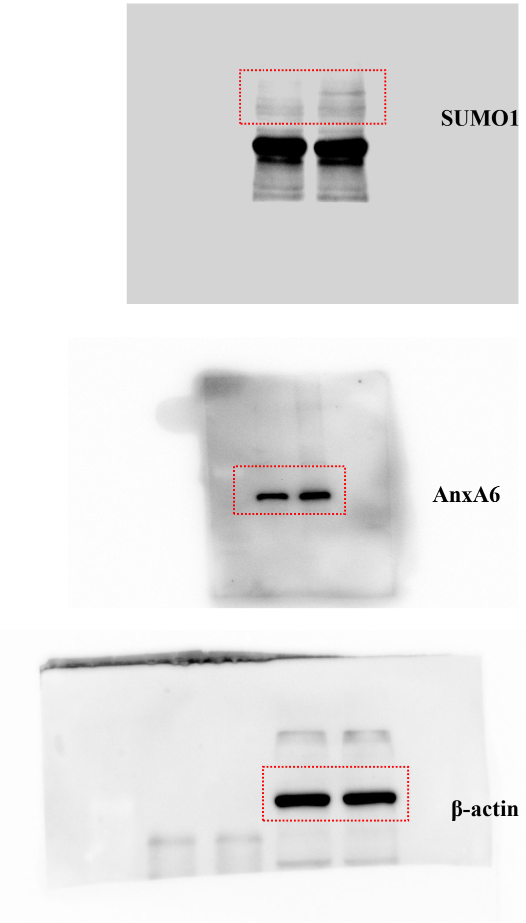

Figure 4B

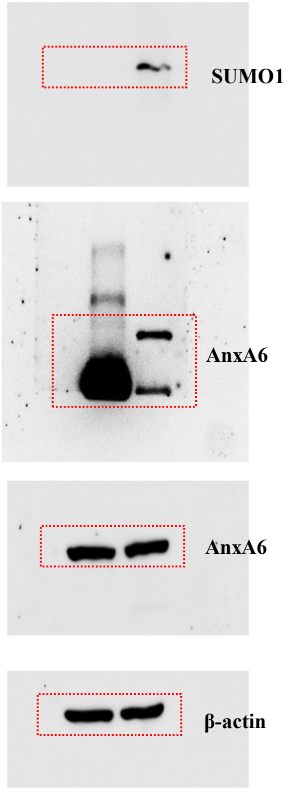

Figure 4C

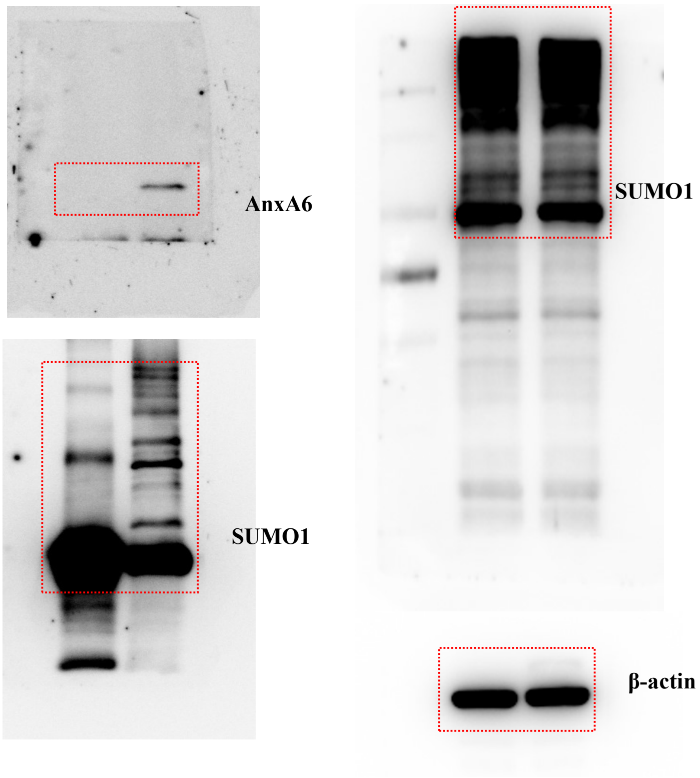

Figure 4D

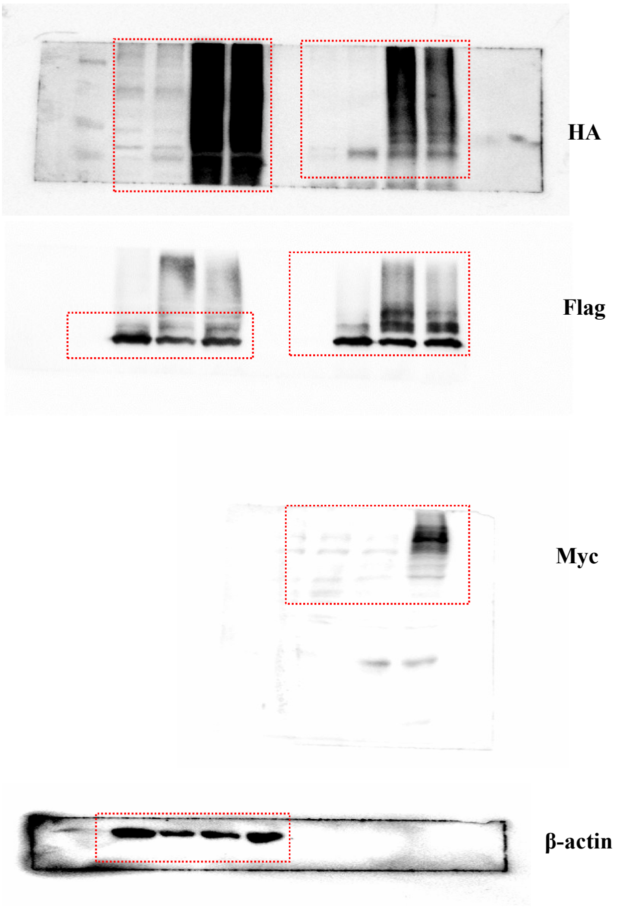

Figure 4F

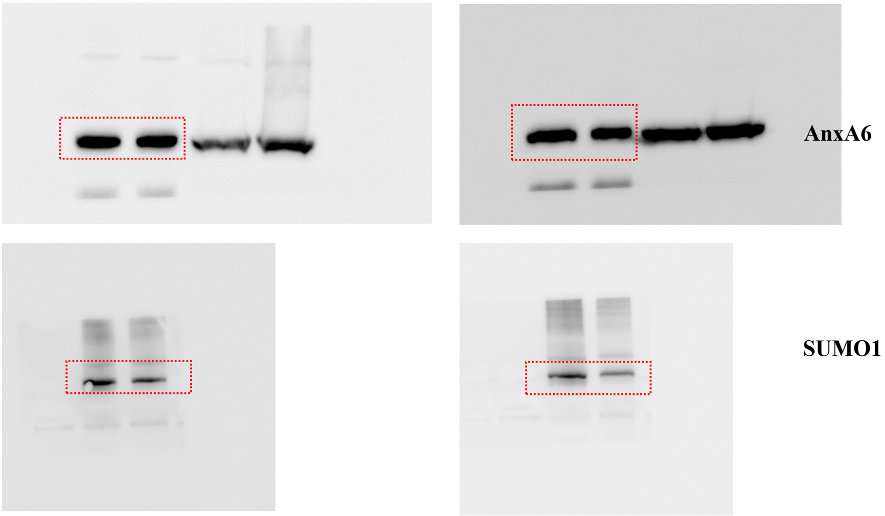

Figure 4G

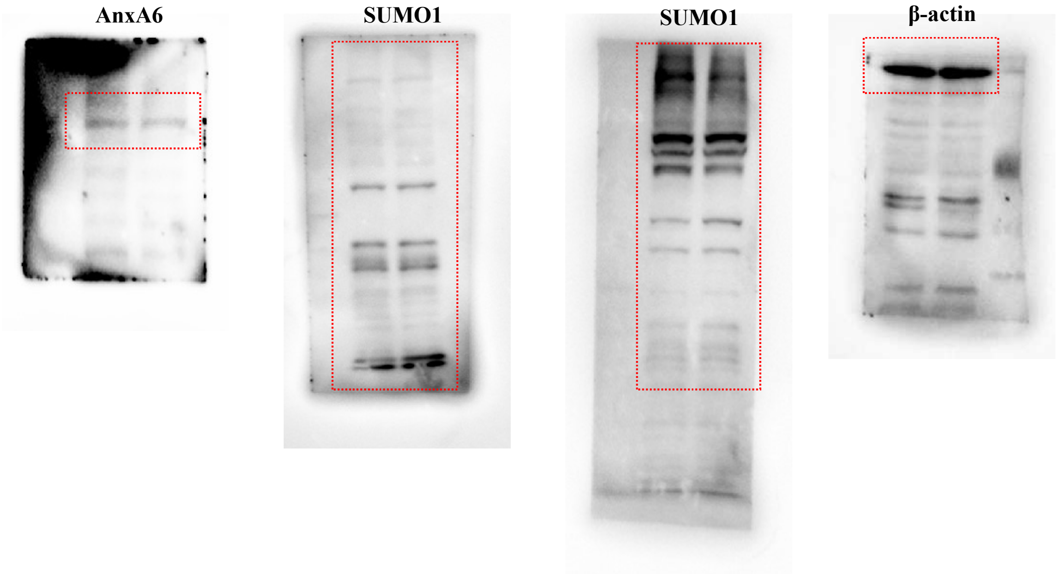

Figure 4H

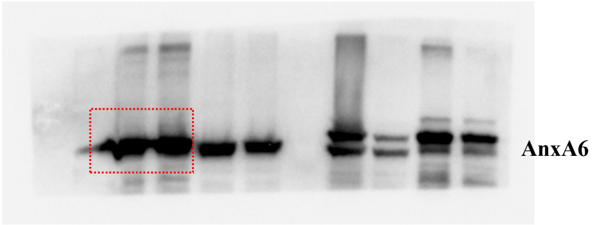

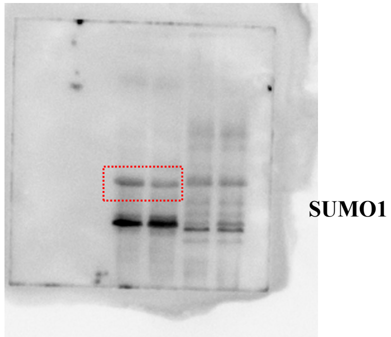

Figure 5C

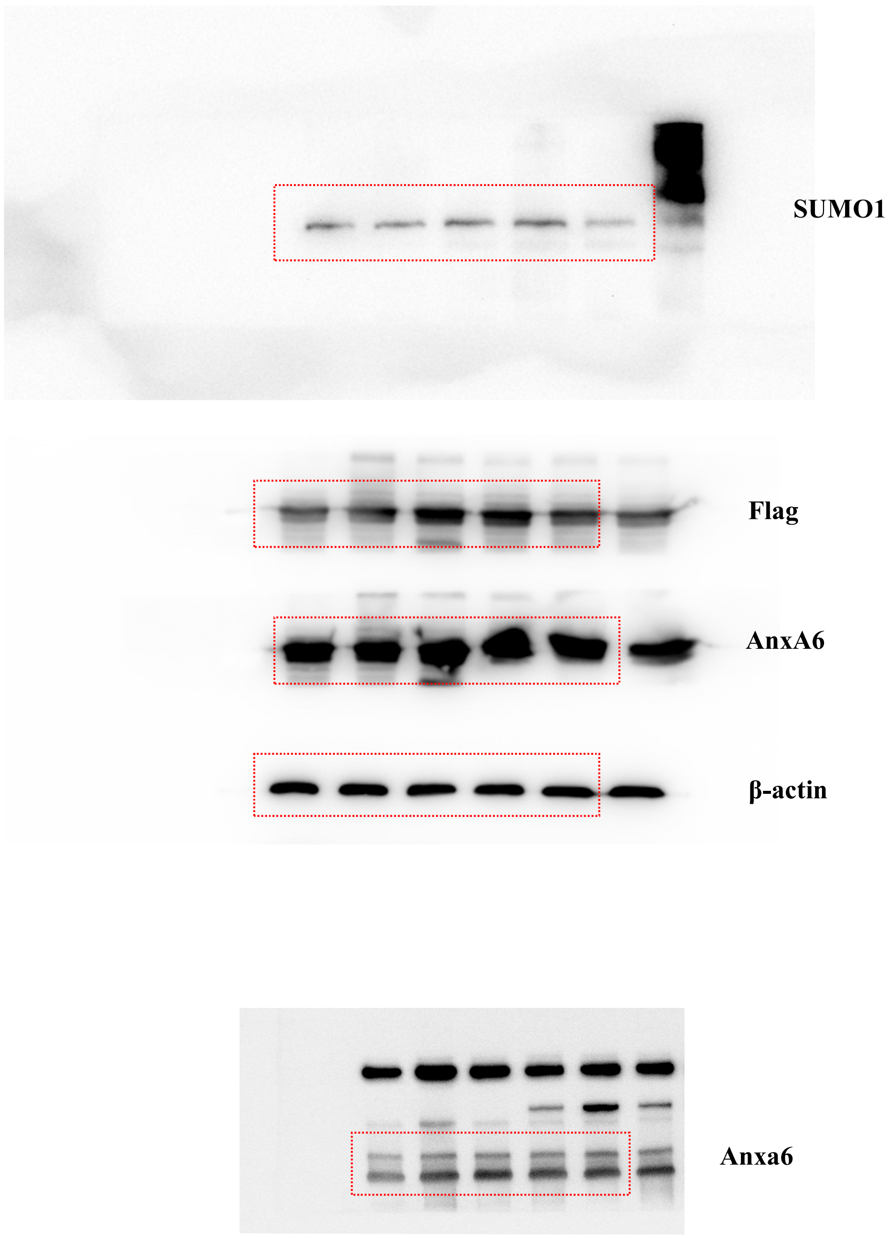

Figure 5D

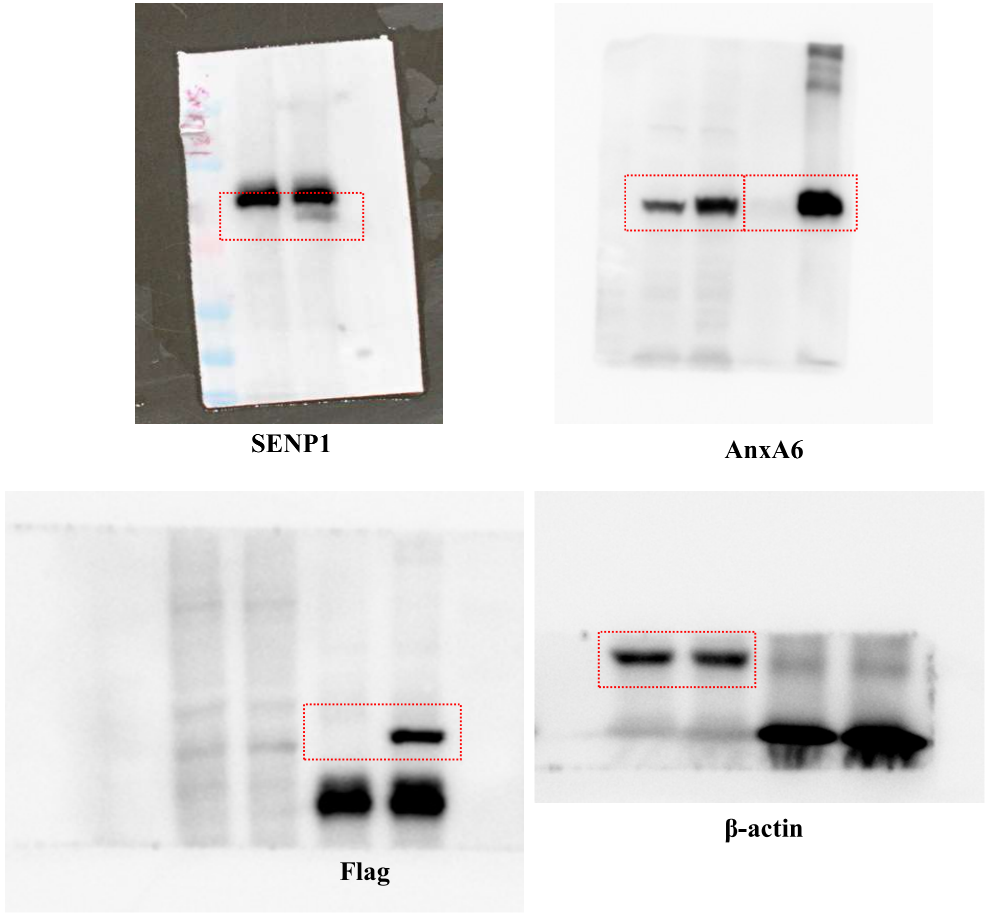

Figure 5E

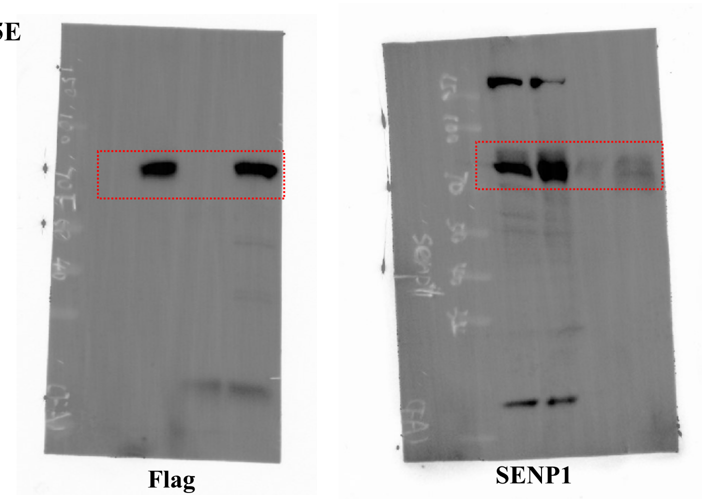

Figure 5F

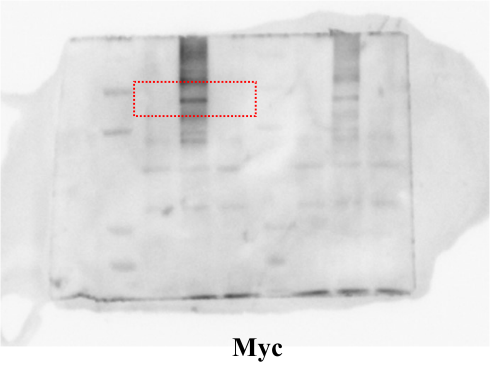

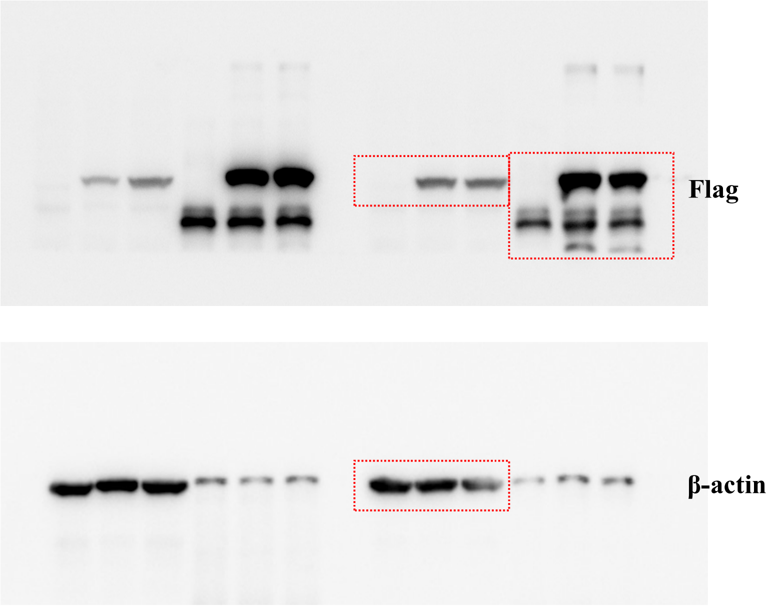

Figure 6A

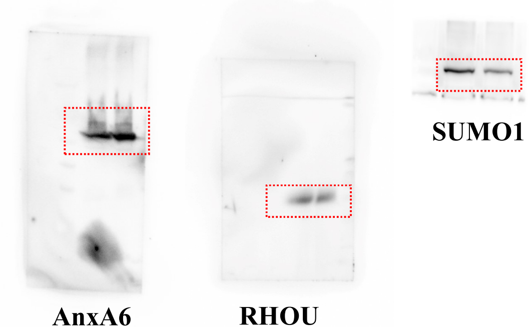

Figure 6C

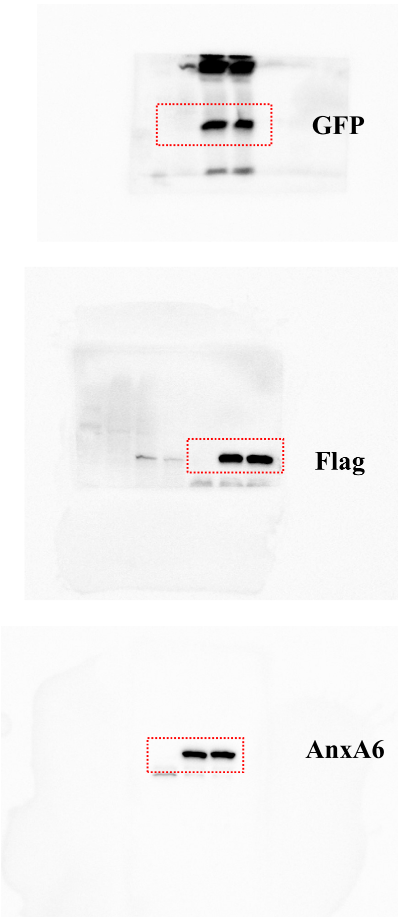

Figure 6B

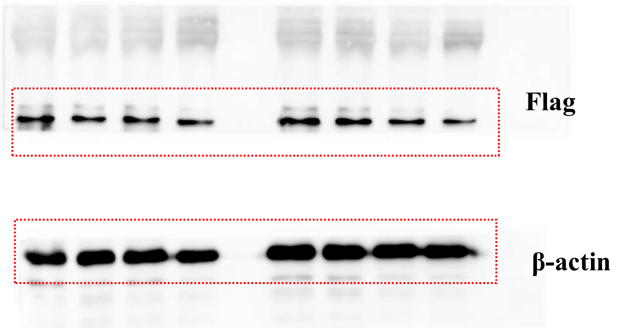

Figure 6D

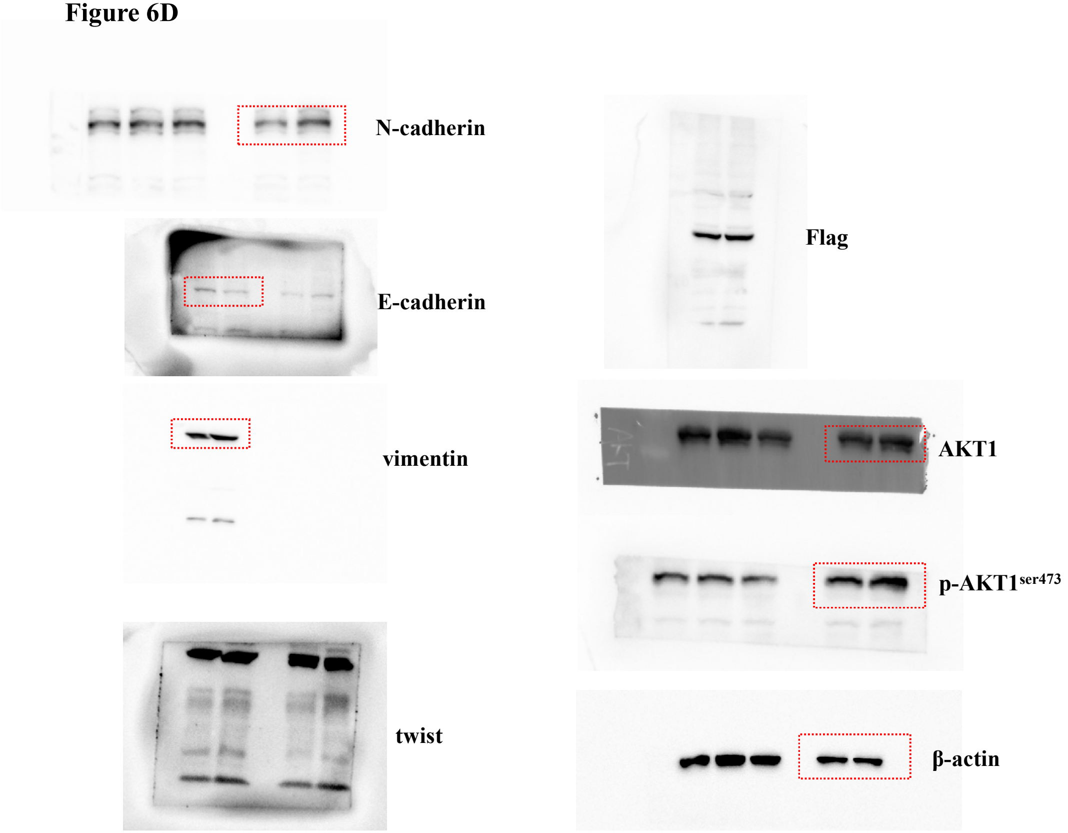
N-cadherin
E-cadherin
vimentin
twist
Flag
AKT1
p-AKT1ser473
β-actin
